# ORE-Mamba: Objective-Driven Representation Evolution with Mamba for Point Cloud Analysis

**DOI:** 10.3390/s26217071

**Published:** 2026-11-09

**Authors:** Yuan Huang, Xiaoyang Wang, Jiayu Zhang, Shuhan Chen, Yu Zhao

**Affiliations:** College of Information and Artificial Intelligence, Yangzhou University, Yangzhou 225127, China; mz120240978@stu.yzu.edu.cn (X.W.); mz120240993@stu.yzu.edu.cn (J.Z.); shchen@yzu.edu.cn (S.C.); zhaoyu@yzu.edu.cn (Y.Z.)

**Keywords:** point cloud representation learning, objective-driven representation evolution, state space models, Mamba, local geometry modeling, receptive-field attention

## Abstract

**Highlights:**

**What are the main findings?**

- ORE-Mamba introduces objective-driven representation evolution, enabling geometry-oriented and saliency-oriented features to evolve through distinct yet collaborative pathways rather than a single shared representation.
- Extensive experiments on ModelNet40, ScanObjectNN, and ShapeNetPart demonstrate the effectiveness of ORE-Mamba across classification, part segmentation, and few-shot recognition, achieving 95.24% and 95.83% accuracy on ScanObjectNN OBJ-BG without and with pre-training, respectively.

**What are the implications of the main findings?**

- Maintaining distinct yet interacting representation trajectories provides an effective alternative to unified feature evolution for learning structure-aware and task-discriminative point-cloud representations.
- The proposed framework provides a promising representation-learning approach for AI-enabled 3D sensing and intelligent point-cloud perception, with potential applications in robotic and other sensor-based systems.

**Abstract:**

Point clouds are a fundamental sensing modality for object perception, scene understanding, and robotic interaction in modern three-dimensional sensing systems. Effective point-cloud understanding requires representations that preserve intrinsic geometric structure while progressively emphasizing task-discriminative information. However, existing Point Mamba architectures typically encode these heterogeneous requirements in a unified token state that evolves along a shared state-space trajectory, potentially limiting functional specialization and complementarity. To address this limitation, we propose ORE-Mamba, an objective-driven representation evolution framework that organizes feature transformations according to their intended functional roles. Here, objective-driven refers to the functional purpose assigned to a representation pathway rather than to an additional training objective or auxiliary loss. ORE-Mamba maintains geometry-oriented and saliency-oriented representations in two distinct but collaborative streams. The geometry stream employs Local Geometry Aggregation Mamba (LGAM) to encode Euclidean neighborhood relations and propagate geometry-aware information over long ranges through a projected-channel bidirectional state-space model (Bi-SSM). The saliency stream employs Receptive-Field Coordinate Attention (RFCA), which combines Morton Z-order serialization with receptive-field-aware position–channel recalibration to selectively enhance task-relevant responses. Stage-wise bidirectional residual interaction exchanges complementary information between the two streams, while token-wise gated fusion adaptively integrates their final representations. Experiments on ModelNet40, ScanObjectNN, and ShapeNetPart demonstrate strong performance in object classification, part segmentation, and few-shot recognition. Without pre-training, ORE-Mamba achieves mean accuracies of 94.0% on ModelNet40 and 95.24%, 94.03%, and 92.11% on the OBJ-BG, OBJ-ONLY, and PB-T50-RS variants of ScanObjectNN, respectively, across three independent training runs. With 15.7 M parameters and 4.7 G FLOPs, ORE-Mamba provides a favorable trade-off between representational capacity and computational cost. These results support objective-driven representation evolution as an effective alternative to unified feature propagation for point-cloud learning.

## 1. Introduction

Modern three-dimensional sensing systems acquire point clouds for object perception, scene understanding, and robotic interaction, supporting applications such as infrastructure inspection [1] and agricultural automation [2]. Under practical sensing conditions, point-cloud representations must preserve intrinsic geometry while progressively emphasizing task-discriminative information for downstream prediction [3,4]. Unlike regular image grids, however, point clouds are irregular, unordered, and spatially sparse, and therefore lack predefined neighborhoods for propagating geometric relations and task-relevant information. The challenge is therefore not only to extract expressive features, but also to organize their geometric and discriminative refinement across network depth.

Point-cloud representation learning has evolved from point-based and graph-based architectures to Transformer-based and, more recently, state-space-based approaches [3,5,6,7,8,9]. Early methods established permutation-invariant point processing and hierarchical neighborhood aggregation [3,5], while graph neural networks and Transformers further strengthened local interaction and global dependency modeling [6,7]. More recently, structured state-space models (SSMs), particularly Mamba, have provided an efficient alternative for long-range representation propagation with computational complexity linear in sequence length [9]. Building on this advantage, Point Mamba methods have investigated point serialization, local geometry enhancement, scan-oriented propagation, and structure-aware state modeling for unordered 3D data [10,11,12,13,14]. Despite their diverse serialization and token-mixing strategies, these methods generally merge the processed information into a common latent state that is propagated through the subsequent network stages.

A common latent trajectory requires the same representation to preserve geometric relations and develop task-discriminative responses, although these two functional requirements favor different transformations. Geometry-oriented representations benefit from explicit Euclidean neighborhoods, relative spatial relations, and structure-preserving aggregation. In contrast, task-discriminative representations require input-dependent selection that emphasizes informative positions and feature channels while suppressing less relevant responses. Here, saliency refers to this task-dependent concentration of discriminative responses rather than externally supervised visual saliency. Propagating both requirements through a uniform evolution trajectory may cause functional interference and restrict the degree to which complementary representation roles can specialize. This observation raises a central question: *Should a shared point-cloud representation be required to satisfy different functional requirements through a uniform evolution trajectory, or should role-specific representations evolve through distinct yet collaborative transformations?*

**Figure 1:**
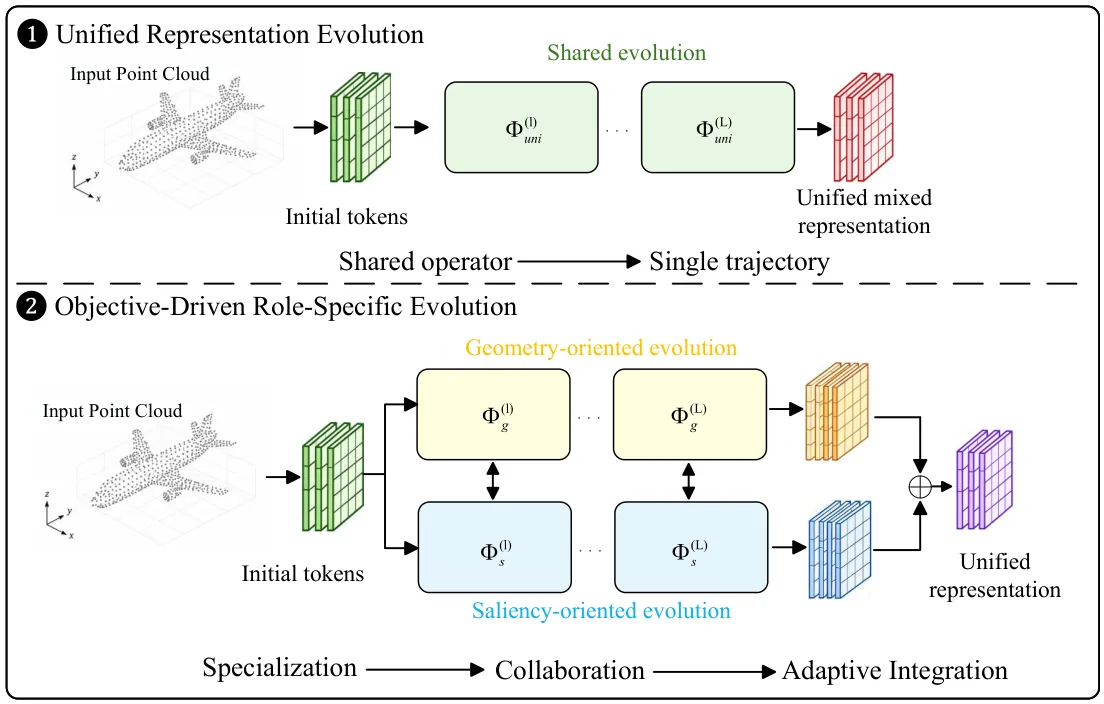
Conceptual comparison between unified representation evolution and objective-driven role-specific evolution. In a representative unified paradigm, heterogeneous functional requirements are consolidated into a common latent state and propagated through a shared representation trajectory, which may cause functional interference and limit role specialization. ORE-Mamba instead initializes two streams from the same point-token representation and progressively induces geometry-oriented and saliency-oriented roles through distinct yet collaborative evolution pathways, followed by adaptive integration.

As conceptually illustrated in Figure 1, these two paradigms embody different assumptions about how point-cloud representations should be progressively refined.

To investigate this question, we introduce *objective-driven representation evolution*, a design principle that organizes feature transformations according to their intended functional roles. Here, objective-driven denotes the functional purpose assigned to a representation pathway rather than an additional training objective or auxiliary loss. Under this principle, representations serving different roles are allowed to specialize through dedicated evolution mechanisms, exchange complementary information during network propagation, and integrate only when a unified task representation is required. We instantiate this principle using a compact two-role design comprising geometry-oriented evolution for preserving and enriching structural information and saliency-oriented evolution for concentrating task-discriminative responses. Both streams are initialized from the same point-token sequence; their specialization is progressively induced by different evolution operators rather than by explicit feature decomposition or separate supervision. Stage-wise communication then enables complementary evidence exchange without immediately collapsing the two role-specific states.

Following this principle, we develop ORE-Mamba, an objective-driven representation evolution architecture for point-cloud analysis. Its geometry stream employs Local Geometry Aggregation Mamba (LGAM), which injects explicit Euclidean neighborhood relations before long-range evolution through a projected-channel Bi-SSM. Its saliency stream employs Receptive-Field Coordinate Attention (RFCA), which organizes irregular patch tokens using Morton Z-order serialization and applies receptive-field-aware position–channel recalibration. This mechanism complements LGAM’s explicit Euclidean neighborhood aggregation by allowing sequence windows to incorporate patches beyond LGAM’s direct neighborhoods. Because independently evolving streams may otherwise become isolated, ORE-Mamba introduces stage-wise bidirectional residual interaction to exchange complementary information while preserving their distinct transformation behaviors. A token-wise adaptive gate finally determines the relative contribution of the two evolved states and integrates them into a unified task-oriented representation.

**Figure 2:**
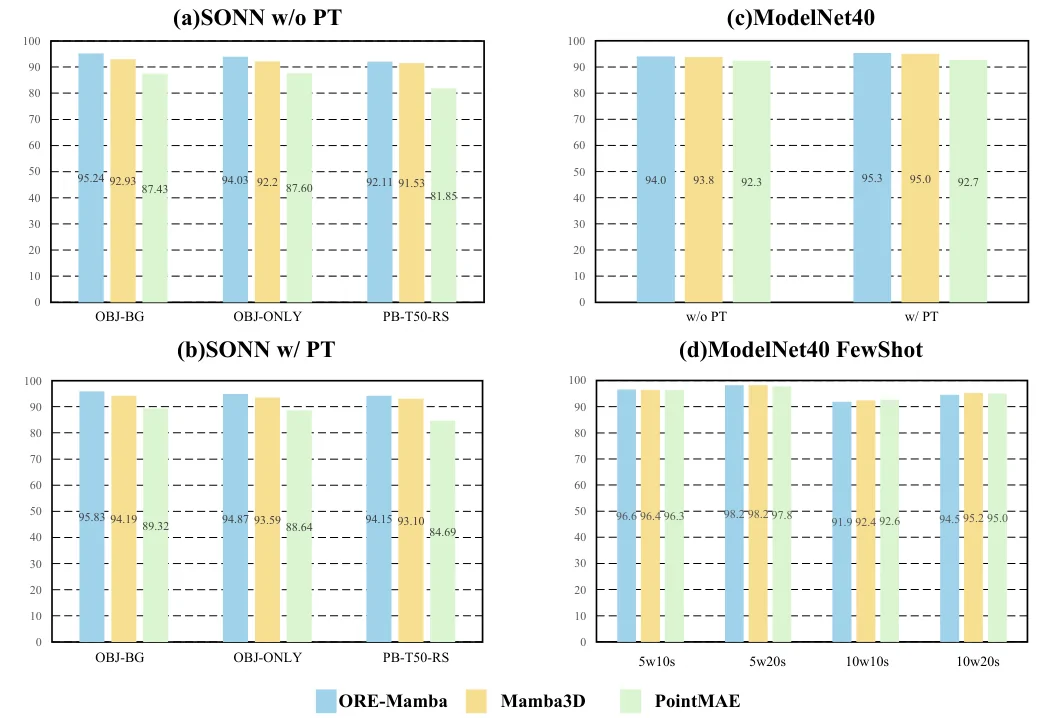
Comparison with existing methods across different tasks. On ScanObjectNN without pre-training (**a**), ORE-Mamba achieves 95.24% on OBJ-BG, and with pre-training (**b**) it reaches 95.83% on OBJ-BG, both representing state-of-the-art performance among the single-model methods considered. On ModelNet40 (**c**), the proposed method also achieves competitive performance. On the ModelNet40 few-shot task (**d**), although it does not achieve the best result in every setting, it demonstrates promising transfer capability.

We evaluate ORE-Mamba on ModelNet40, ScanObjectNN, and ShapeNetPart across object classification, part segmentation, and few-shot recognition. Comparative experiments and ablation studies assess the contributions of the architectural components and their interaction. In particular, ORE-Mamba achieves a mean accuracy of 95.24% on the OBJ-BG split of ScanObjectNN without pre-training and 95.83% accuracy with pre-training, as shown in Figure 2.

The main contributions of this work are summarized as follows:

- We revisit the unified representation evolution paradigm commonly adopted in Point Mamba architectures and highlight a potential limitation of propagating heterogeneous functional requirements through a single latent trajectory. Based on this observation, we introduce *objective-driven representation evolution*, a design principle that organizes stage-wise feature transformations according to their intended functional roles rather than propagating all information through a uniform evolution process.
- We propose ORE-Mamba, a dual-stream architecture that instantiates the proposed principle through persistent geometry-oriented and saliency-oriented representation evolution. Both streams are initialized from the same point-token sequence but are progressively specialized by dedicated operators. Local Geometry Aggregation Mamba (LGAM) couples explicit Euclidean neighborhood aggregation with a Bi-SSM in the projected-channel space, whereas Receptive-Field Coordinate Attention (RFCA) establishes a serialization-aware saliency evolution mechanism that selectively recalibrates task-relevant responses across serialized positions and expanded feature channels.
- We develop a collaborative evolution mechanism consisting of stage-wise bidirectional residual interaction and token-wise adaptive gated fusion. The interaction mechanism enables complementary evidence to be exchanged across network depth without prematurely collapsing the two role-specific representation states, while the adaptive gate determines their token-dependent contributions to the final unified task representation.

## 2. Related Work

### 2.1. Point Cloud Representation Learning

Point-cloud representation learning has progressed through several architectural paradigms. PointNet [3] established permutation-invariant processing of unordered point sets, and PointNet++ [5] introduced hierarchical neighborhood aggregation for multi-scale local modeling. Graph- and convolution-based methods subsequently strengthened geometry-aware interaction: DGCNN [6] dynamically constructs feature-space graphs, PointConv [15] parameterizes continuous kernels from relative coordinates, KPConv [16] places learnable kernel points in Euclidean space, PAConv [17] assembles position-adaptive kernels, and RepSurf [18] encodes fine-grained surface priors. Lightweight architectures such as PointMLP [19] and PointNeXt [8] further demonstrated that careful architectural scaling and training design can yield strong point representations.

Attention-based methods provide content-dependent alternatives to fixed local aggregation. Point Transformer [7] introduced position-aware vector attention, Point Transformer V2 [20] improved this formulation through grouped vector attention and partition-based pooling, and PointMixer [21] generalized feature mixing across intra-set, inter-set, and hierarchical relations. Point Transformer V3 [22] replaced expensive precise neighbor search with serialized neighborhood mapping, enabling efficient scaling to larger receptive fields. In parallel, self-supervised methods such as Point-BERT [23], Point-MAE [24], Point-M2AE [25], Point2Vec [26], I2P-MAE [27], PointGPT [28], Point-JEPA [29], and Sonata [30] have improved transferability through masked prediction, reconstruction, autoregressive generation, and self-distillation.

These studies have substantially improved local geometric encoding, global interaction, architectural scalability, and supervisory design. The present work focuses on a different question: how representations serving heterogeneous functional roles should be maintained and transformed throughout network depth.

### 2.2. State Space Models for Point Clouds

Structured State Space Models (SSMs) were developed as efficient sequence models that propagate information through recurrent latent-state transitions [31]. Mamba [9] further introduced input-dependent selective state spaces, enabling content-aware long-range modeling with complexity linear in sequence length. These properties have motivated a rapidly growing body of work that adapts Mamba to irregular point-cloud data.

PointMamba [10] converts point patches into ordered sequences using space-filling curves and employs a non-hierarchical Mamba encoder for efficient global propagation. Point Cloud Mamba [12] introduces consistent traversal serialization, order-aware prompts, and coordinate-based positional encoding to improve the compatibility between unordered points and causal sequence modeling. Mamba3D [11] combines Local Norm Pooling with bidirectional state-space modeling, whereas Pamba [13] uses multi-path serialization and ConvMamba blocks to strengthen local geometry modeling and global interaction. More recent methods further incorporate graph spectral information, spatial states, sequence expansion, chained propagation, and grouped selective state spaces [14,32,33].

Existing Point Mamba studies have therefore explored serialization order, local enhancement, bidirectional modeling, spectral structure, spatial-state preservation, and selective-state parameterization. Some methods process multiple traversal orders or combine heterogeneous operators, but the resulting information is generally merged into a common latent state for subsequent propagation. ORE-Mamba instead investigates whether different functional requirements should be preserved as persistent role-specific representations and evolved through dedicated yet collaborative pathways.

### 2.3. Geometry-Aware and Selective Information Modeling

Accurate point-cloud understanding depends on both the preservation of intrinsic geometric relations and the selective amplification of task-relevant responses. Geometry-aware methods encode local structures through hierarchical neighborhoods [5], dynamic graphs [6], continuous or kernel-point convolutions [15,16], adaptive kernel assembly [17], and local surface descriptors [18]. These mechanisms improve sensitivity to relative coordinates, neighborhood shapes, and fine-grained surface variations.

Selective aggregation methods learn input-dependent weights for emphasizing informative responses. Point Transformer [7] and Point Transformer V2 [20] modulate local interactions through position-aware attention, PointMixer [21] learns relation-dependent mixing weights, and Point Transformer V3 [22] demonstrates that serialized neighborhoods can efficiently enlarge contextual interaction. Related mechanisms in regular visual domains also provide useful principles. Coordinate Attention [34] embeds positional information into channel recalibration, while Receptive-Field Attention Convolution [35] assigns adaptive weights within expanded convolutional receptive fields. In this work, saliency denotes the input-dependent concentration of task-discriminative responses rather than externally supervised visual saliency.

Geometry-aware aggregation and selective recalibration are typically used to improve a single shared feature state. Their complementary functions are therefore combined within the same update trajectory. ORE-Mamba instead associates them with persistent geometry-oriented and saliency-oriented representation roles that evolve through different transformation mechanisms.

### 2.4. Multi-Operator and Structured Representation Modeling

Many point-cloud architectures combine heterogeneous operators or multiple processing paths to exploit complementary modeling properties. Local geometric modeling is combined with attention- or state-space-based propagation in Point Transformer [7], Mamba3D [11], and Pamba [13]. Other methods introduce different serialization orders or parallel paths to capture complementary spatial information [10,12,13,33,36]. For example, DuoMamba [36] employs two parallel Mamba streams constructed from different space-filling curves, providing complementary spatial observations through traversal diversity.

Structured decomposition has also been explored across data dimensions and network stages. Mamba4D [37] separates spatial and temporal modeling in point-cloud videos through dedicated intra-frame and inter-frame SSM modules. LitePT [38] assigns convolutional processing to early high-resolution stages for local geometric modeling and attention to deeper low-resolution stages for semantic interaction. These designs demonstrate the value of matching processing mechanisms to different dependency types and representation characteristics.

ORE-Mamba shares the motivation of exploiting complementary transformations, but organizes its pathways around geometric modeling and task-discriminative response selection within a static point-cloud encoder. Its two states remain separately propagated across stages, exchange information through bidirectional interaction, and undergo adaptive integration at the final stage. This organization differs from diversifying traversal orders, separating spatial and temporal dependencies, or assigning different operators to successive network stages. Table 1 summarizes how representative point-cloud Mamba methods organize local geometry modeling, serialization, and feature states. In particular, it distinguishes temporary traversal or state-space branches from role-specific representations that persist across stages. The comparison concerns architectural organization rather than accuracy across the different tasks addressed by these methods.

### 2.5. Discussion

The reviewed literature advances point-cloud representation learning through geometric aggregation, scalable propagation, and adaptive feature selection. A complementary question is how these functions should be organized throughout network depth. ORE-Mamba investigates persistent functional specialization with cross-stream communication, building on these established modeling mechanisms. The following section formalizes this organization and describes its implementation; the ablation studies subsequently assess the contributions of the individual components and their interaction.

## 3. Method

This section first formulates objective-driven representation evolution and then describes its implementation in ORE-Mamba, including the two evolution pathways, cross-stream interaction, and adaptive integration.

Unless otherwise specified, the batch dimension is omitted. Bold uppercase symbols denote token matrices, bold lowercase symbols denote vectors, and calligraphic symbols denote sets or abstract operators. In an expression such as [a;B], the semicolon denotes concatenation along the token dimension. Feature-channel concatenation is written explicitly as ${\mathrm{Concat}}_{\mathrm{ch}}\left(\;\cdot \;\right)$.

### 3.1. Objective-Driven Representation Evolution

We now formalize objective-driven representation evolution using two persistent representation states. The geometry-oriented state emphasizes structural relations, while the saliency-oriented state emphasizes task-discriminative responses. At each stage, dedicated operators update the two states, followed by cross-stream interaction; adaptive integration produces the final task representation. This two-role design provides a tractable instantiation of the proposed principle without assuming an exhaustive decomposition of point-cloud representation functions.

Let $F_{g}^{\left(l-1\right)}$ and $F_{s}^{\left(l-1\right)}$ denote the geometry-oriented and saliency-oriented representations entering the *l*-th evolution stage, respectively, and let $C\in R^{M\times 3}$ contain the patch-center coordinates. The two representations first undergo role-specific evolution:

(1) $$\begin{array}{cc} U_{g}^{\left(l\right)}\; & =E_{g}^{\left(l\right)}\left(F_{g}^{\left(l-1\right)};C\right),\\ U_{s}^{\left(l\right)}\; & =E_{s}^{\left(l\right)}\left(F_{s}^{\left(l-1\right)};C\right), \end{array}$$

where $E_{g}^{\left(l\right)}$ and $E_{s}^{\left(l\right)}$ denote geometry-oriented and saliency-oriented evolution operators, respectively. The coordinate matrix provides explicit Euclidean relations for geometry evolution and spatial organization cues for saliency evolution.

Independent specialization alone may isolate the two representations and prevent either stream from exploiting information emphasized by the other. The independently evolved states therefore exchange complementary information through a collaborative interaction operator:

(2) $$\left(F_{g}^{\left(l\right)},F_{s}^{\left(l\right)}\right)=L_{i}^{\left(l\right)}\left(U_{g}^{\left(l\right)},U_{s}^{\left(l\right)}\right).$$

Importantly, intermediate interaction does not explicitly merge the two representations into a single latent state. Instead, each stream is updated with information projected from the opposite stream while retaining its own functional trajectory. Geometry evolution can therefore benefit from task-discriminative evidence, whereas saliency evolution remains informed by structure-aware cues.

After *L* evolution stages, the two collaboratively evolved representations are integrated into a unified task-oriented representation:

(3) $$F_{\mathrm{out}}=L_{f}\left(F_{g}^{\left(L\right)},F_{s}^{\left(L\right)}\right),$$

where $L_{f}$ denotes an adaptive integration operator. Interaction and integration serve different purposes: stage-wise interaction exchanges complementary information while preserving role-specific evolution, whereas final integration terminates the dual-stream process and determines the relative contributions of the two representations for downstream prediction.

The following sections instantiate these abstract operators while preserving this specialization–collaboration–integration hierarchy.

### 3.2. ORE-Mamba Architecture

**Figure 3:**
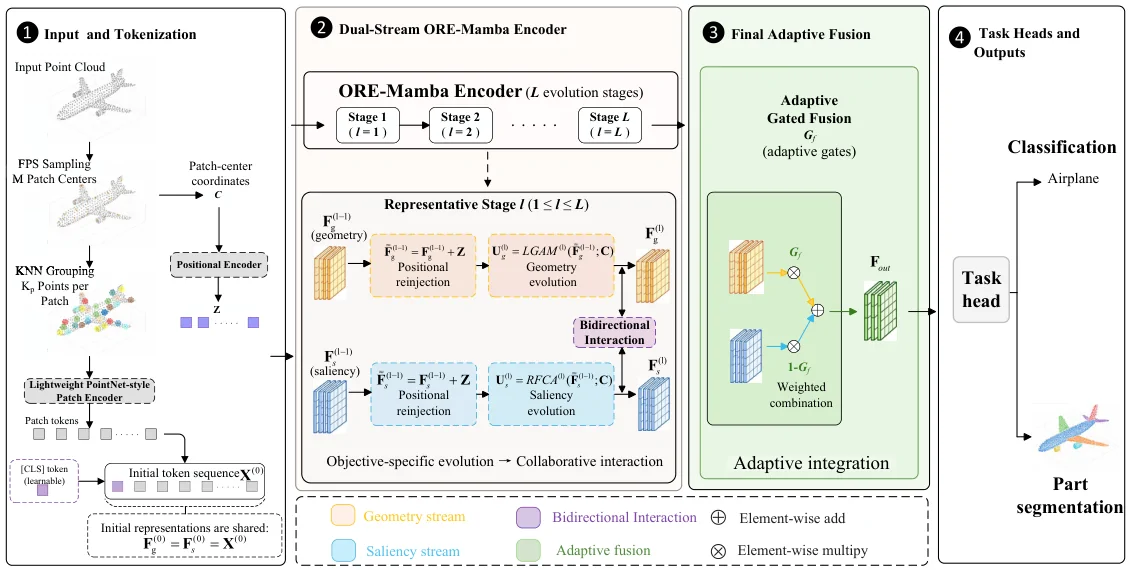
Overall architecture of ORE-Mamba. The input point cloud is partitioned into *M* local patches through FPS and KNN grouping and encoded into the initial token sequence $X^{\left(0\right)}$. Geometry-oriented and saliency-oriented representations are initialized from the same tokens and evolved over *L* stages by LGAM and RFCA, respectively. At each stage, synchronous bidirectional residual interaction exchanges complementary information while preserving two role-specific evolution trajectories. The final normalized representations are integrated through a token-wise adaptive gate to produce the unified representation $F_{\mathrm{out}}$, which is fed to task-specific heads for object classification or part segmentation. Orange, blue, purple, and green denote geometry evolution, saliency evolution, cross-stream interaction, and adaptive fusion, respectively.

Following the objective-driven representation evolution principle, ORE-Mamba instantiates geometry-oriented and saliency-oriented evolution using Local Geometry Aggregation Mamba (LGAM) and Receptive-Field Coordinate Attention (RFCA), respectively. At each stage, the two streams first undergo role-specific evolution and subsequently exchange complementary information through synchronous bidirectional interaction. After the final stage, token-wise adaptive gated fusion integrates the collaboratively evolved representations into a unified representation for downstream prediction. The overall architecture is illustrated in Figure 3, and its stage-wise forward computation is summarized in Algorithm 1.

The assignment of LGAM and RFCA to the two streams is motivated by their complementary inductive biases. LGAM constructs Euclidean neighborhoods among patch centers and encodes relative coordinates and neighbor-to-center feature differences before local aggregation. These operations make local spatial structure explicit, while the subsequent Bi-SSM propagates the geometry-enriched features across patch tokens. LGAM is therefore suited to geometry-oriented evolution. RFCA uses Morton-order serialization to organize patch tokens into sequence windows and applies input-dependent position–channel weights to recalibrate their responses. Since LGAM already explicitly aggregates Euclidean neighbors, RFCA is designed to retain spatial structure while incorporating context beyond those direct neighborhoods. Its recalibration emphasizes responses useful for the downstream task, matching the saliency-oriented role. These roles describe the operators’ intended inductive biases; they do not imply explicitly supervised or strictly disentangled representations.
**Algorithm 1** Forward Computation of ORE-Mamba**Require:** Point cloud $P\in R^{N\times 3}$**Ensure:** Unified representation $F_{\mathrm{out}}$  1:Select patch centers C by FPS and construct centered local patches ${\left\{\Delta G_{i}\right\}}_{i=1}^{M}$ by KNN  2:Encode the local patches into $X_{p}$ and construct $X^{\left(0\right)}=\left[x_{\mathrm{cls}};X_{p}\right]$ and $Z=[z_{\mathrm{cls}};E_{\mathrm{pos}}\left(C\right)]$  3:Initialize $F_{g}^{\left(0\right)}\leftarrow X^{\left(0\right)}$ and $F_{s}^{\left(0\right)}\leftarrow X^{\left(0\right)}$  4:**for** l=1 to *L* **do**  5:      ${\tilde{F}}_{g}^{\left(l-1\right)}\leftarrow F_{g}^{\left(l-1\right)}+Z$  6:      ${\tilde{F}}_{s}^{\left(l-1\right)}\leftarrow F_{s}^{\left(l-1\right)}+Z$  7:      $U_{g}^{\left(l\right)}\leftarrow {\mathrm{LGAM}}^{\left(l\right)}\left({\tilde{F}}_{g}^{\left(l-1\right)};C\right)$  8:      $U_{s}^{\left(l\right)}\leftarrow {\mathrm{RFCA}}^{\left(l\right)}\left({\tilde{F}}_{s}^{\left(l-1\right)};C\right)$  9:      ${\bar{U}}_{g}^{\left(l\right)}\leftarrow {\mathrm{LN}}_{i,g}^{\left(l\right)}\left(U_{g}^{\left(l\right)}\right)$10:      ${\bar{U}}_{s}^{\left(l\right)}\leftarrow {\mathrm{LN}}_{i,s}^{\left(l\right)}\left(U_{s}^{\left(l\right)}\right)$11:      $\Delta U_{s\to g}^{\left(l\right)}\leftarrow P_{s\to g}^{\left(l\right)}\left({\bar{U}}_{s}^{\left(l\right)}\right)$12:      $\Delta U_{g\to s}^{\left(l\right)}\leftarrow P_{g\to s}^{\left(l\right)}\left({\bar{U}}_{g}^{\left(l\right)}\right)$13:      $F_{g}^{\left(l\right)}\leftarrow U_{g}^{\left(l\right)}+\Delta U_{s\to g}^{\left(l\right)}$14:      $F_{s}^{\left(l\right)}\leftarrow U_{s}^{\left(l\right)}+\Delta U_{g\to s}^{\left(l\right)}$15:**end for**16:${\bar{F}}_{g}\leftarrow {\mathrm{LN}}_{f}\left(F_{g}^{\left(L\right)}\right)$ and ${\bar{F}}_{s}\leftarrow {\mathrm{LN}}_{f}\left(F_{s}^{\left(L\right)}\right)$17:$G_{f}\leftarrow \sigma \;\left(\psi_{f}\;\left({\mathrm{Concat}}_{\mathrm{ch}}\left({\bar{F}}_{g},{\bar{F}}_{s}\right)\right)\right)$18:$F_{\mathrm{out}}\leftarrow {\mathrm{LN}}_{f}\;\left(G_{f}⊙{\bar{F}}_{g}+\left(1-G_{f}\right)⊙{\bar{F}}_{s}\right)$19:**return** $F_{\mathrm{out}}$

Given an input point cloud $P={\left\{p_{n}\right\}}_{n=1}^{N}\in R^{N\times 3}$, farthest-point sampling selects *M* patch centers, $C={\left[c_{1},\ldots ,c_{M}\right]}^{T}\in R^{M\times 3}$. For each center $c_{i}$, its $K_{p}$ nearest input points form a centered local patch,

(4) $$\Delta G_{i}=\left\{p_{j}-c_{i}\;|\;j\in \mathrm{KNN}\left(c_{i},P,K_{p}\right)\right\}\in R^{K_{p}\times 3}.$$

A shared PointNet-style encoder maps each patch to a *d*-dimensional token,

(5) $$X_{p}={\mathrm{Stack}}_{i=1}^{M}\left[E_{\mathrm{patch}}\left(\Delta G_{i}\right)\right]\in R^{M\times d}.$$

A learnable prefix token, denoted CLS, $x_{\mathrm{cls}}\in R^{d}$, is prepended to the patch tokens. Positional embeddings are generated from the patch centers, together with a learnable CLS positional embedding $z_{\mathrm{cls}}\in R^{d}$,

(6) $$\begin{array}{cc} X^{\left(0\right)}\; & =\left[x_{\mathrm{cls}};X_{p}\right]\in R^{T\times d},\\ Z\; & =\left[z_{\mathrm{cls}};E_{\mathrm{pos}}\left(C\right)\right]\in R^{T\times d},\;T=M+1. \end{array}$$

The geometry and saliency streams share the same initial token sequence,

(7) $$F_{g}^{\left(0\right)}=F_{s}^{\left(0\right)}=X^{\left(0\right)}.$$

Although identically initialized, the two streams progressively specialize through different evolution operators. Before every role-specific stage, positional information is re-injected as

(8) $${\tilde{F}}_{g}^{\left(l-1\right)}=F_{g}^{\left(l-1\right)}+Z,\;{\tilde{F}}_{s}^{\left(l-1\right)}=F_{s}^{\left(l-1\right)}+Z.$$

The role-specific stage outputs are

(9) $$\begin{array}{cc} U_{g}^{\left(l\right)}\; & ={\mathrm{LGAM}}^{\left(l\right)}\left({\tilde{F}}_{g}^{\left(l-1\right)};C\right),\\ U_{s}^{\left(l\right)}\; & ={\mathrm{RFCA}}^{\left(l\right)}\left({\tilde{F}}_{s}^{\left(l-1\right)};C\right). \end{array}$$

The two role-specific outputs are subsequently refined through synchronous bidirectional residual interaction and propagated as separate states to the next evolution stage. After the final stage, their normalized representations are integrated through token-wise adaptive gated fusion. The exact interaction and integration formulations are presented in Section 3.5, while implementation-aligned details are provided in Appendix A.5.

### 3.3. Geometry-Oriented Evolution with LGAM

**Figure 4:**
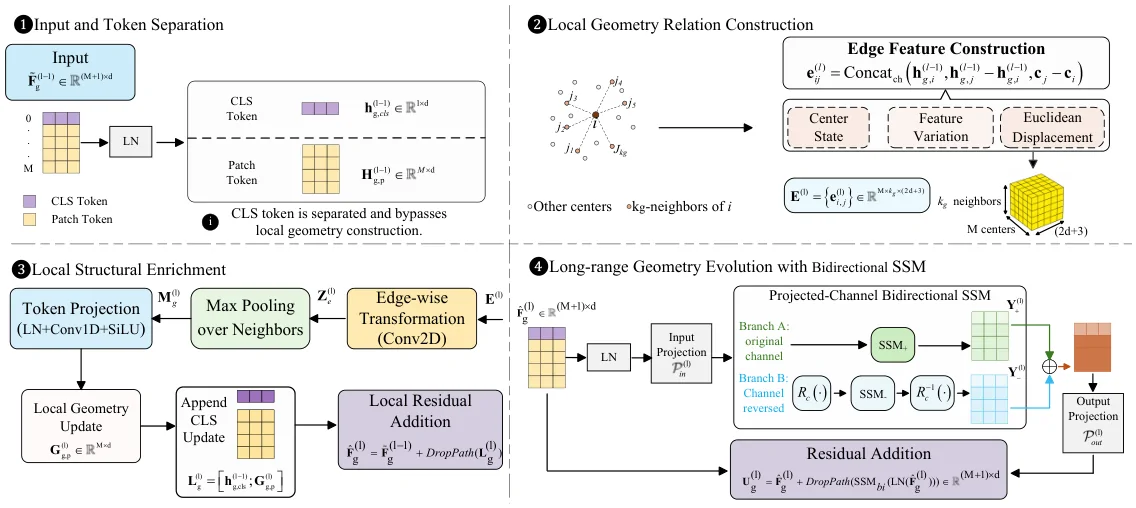
Detailed architecture of the proposed geometry-oriented evolution module based on Local Geometry Aggregation Mamba (LGAM). LGAM first constructs explicit local geometric relations from patch-center neighborhoods and enriches patch representations through edge-wise projection and neighborhood aggregation. The locally enriched representation is subsequently processed by a projected-channel Bi-SSM. One branch processes the original projected feature-channel order, whereas the other processes the reversed projected feature-channel order while preserving the token sequence. The combined branch output is then incorporated through a block-level residual update.

Local Geometry Aggregation Mamba (LGAM) instantiates the geometry-oriented evolution operator. As illustrated in Figure 4, LGAM follows a local-to-global evolution strategy. It first constructs explicit Euclidean relations among neighboring patch centers and aggregates the resulting edge features to enrich each patch token with local structural evidence. The locally enhanced representation is then processed by a projected-channel Bi-SSM, which propagates geometry-aware information beyond the immediate neighborhood. In this manner, explicit local structure is incorporated before long-range representation evolution, rather than being left to emerge implicitly from a one-dimensional token sequence.

At stage *l*, the position-enriched geometry representation is first normalized and partitioned into the classification token and patch tokens,

(10) $$H_{g}^{\left(l-1\right)}={\mathrm{LN}}_{g,1}^{\left(l\right)}\left({\tilde{F}}_{g}^{\left(l-1\right)}\right)=\left[h_{g,\mathrm{cls}}^{\left(l-1\right)};H_{g,p}^{\left(l-1\right)}\right]\in R^{T\times d}.$$

Here, $H_{g,p}^{\left(l-1\right)}=\left[h_{g,1}^{\left(l-1\right)};\ldots ;h_{g,M}^{\left(l-1\right)}\right]\in R^{M\times d}$, where $h_{g,i}^{\left(l-1\right)}\in R^{d}$ denotes the normalized representation of the *i*-th patch token.

Because the classification token has no physical patch center, Euclidean neighborhood construction is performed only for the *M* patch tokens. For patch *i*, LGAM selects the $k_{g}$ nearest *other* patch centers and forms $N_{k_{g}}\left(i\right)$. For each $j\in N_{k_{g}}\left(i\right)$, the center–neighbor relation is represented as

(11) $$e_{ij}^{\left(l\right)}={\mathrm{Concat}}_{\mathrm{ch}}\left(h_{g,i}^{\left(l-1\right)},h_{g,j}^{\left(l-1\right)}-h_{g,i}^{\left(l-1\right)},c_{j}-c_{i}\right)\in R^{2d+3}.$$

where $c_{i}\in R^{3}$ denotes the center coordinate of patch *i*.

The center feature provides the local reference state, the feature difference captures neighbor-dependent variation, and the relative coordinate explicitly encodes Euclidean displacement. A shared edge-wise projection transforms each relation independently,

(12) $$z_{ij}^{\left(l\right)}=\phi_{e}^{\left(l\right)}\left(e_{ij}^{\left(l\right)}\right)\in R^{2d},$$

and channel-wise maximum aggregation over the neighborhood produces

(13) $$m_{g,i}^{\left(l\right)}=\max_{j\in N_{k_{g}}\left(i\right)}z_{ij}^{\left(l\right)}\in R^{2d}.$$

The aggregated response is normalized, projected back to the token dimension, and activated,

(14) $$g_{g,i}^{\left(l\right)}=\mathrm{SiLU}\left(\phi_{g}^{\left(l\right)}\left({\mathrm{LN}}_{e}^{\left(l\right)}\left(m_{g,i}^{\left(l\right)}\right)\right)\right)\in R^{d},$$

where $\phi_{e}^{\left(l\right)}$ and $\phi_{g}^{\left(l\right)}$ are implemented by shared 1×1 edge and token projections, respectively. Stacking the responses of all patch centers gives

(15) $$G_{g,p}^{\left(l\right)}={\mathrm{Stack}}_{i=1}^{M}\left[g_{g,i}^{\left(l\right)}\right]\in R^{M\times d}.$$

The normalized classification token is not geometrically aggregated, but it is retained in the local branch. The local geometry response is therefore reattached to the classification-token response and added to the position-enriched stage input,

(16) $$\begin{array}{cc} L_{g}^{\left(l\right)}\; & =\left[h_{g,\mathrm{cls}}^{\left(l-1\right)};G_{g,p}^{\left(l\right)}\right]\in R^{T\times d},\\ {\hat{F}}_{g}^{\left(l\right)}\; & ={\tilde{F}}_{g}^{\left(l-1\right)}+\mathrm{DropPath}\left(L_{g}^{\left(l\right)}\right). \end{array}$$

Equation (16) is the local residual update, which preserves the incoming token representation while injecting the newly extracted Euclidean neighborhood response. LGAM then performs long-range geometry evolution using a second pre-normalized residual sublayer. Let

(17) $$H_{m}^{\left(l\right)}={\mathrm{LN}}_{g,2}^{\left(l\right)}\left({\hat{F}}_{g}^{\left(l\right)}\right),\;V^{\left(l\right)}=P_{\mathrm{in}}^{\left(l\right)}\left(H_{m}^{\left(l\right)}\right),$$

where $P_{\mathrm{in}}^{\left(l\right)}$ is the shared Mamba input projection. The projected-channel Bi-SSM processes the original and reversed projected feature-channel orders,

(18) $$\begin{array}{cc} Y_{+}^{\left(l\right)}\; & ={\mathrm{SSM}}_{+}^{\left(l\right)}\left(V^{\left(l\right)}\right),\\ Y_{-}^{\left(l\right)}\; & =R_{c}^{-1}\left[{\mathrm{SSM}}_{-}^{\left(l\right)}\left(R_{c}\left(V^{\left(l\right)}\right)\right)\right],\\ B^{\left(l\right)}\left(H_{m}^{\left(l\right)}\right)\; & =P_{\mathrm{out}}^{\left(l\right)}\left(Y_{+}^{\left(l\right)}+Y_{-}^{\left(l\right)}\right). \end{array}$$

Following the channel-reversal design of Mamba3D [11], $R_{c}$ reverses the projected feature-channel order without changing the token sequence. Here, Bi-SSM refers to this two-branch projected-channel construction rather than to forward and backward scans of the token sequence. Because both branches retain causal token dependencies, the prepended CLS token cannot receive information from subsequent patch tokens through this mixer. The projected branch sum is incorporated through the outer block-level residual,

(19) $$U_{g}^{\left(l\right)}={\hat{F}}_{g}^{\left(l\right)}+\mathrm{DropPath}\left(B^{\left(l\right)}\left(H_{m}^{\left(l\right)}\right)\right)\in R^{T\times d}.$$

The local and outer residuals operate at different levels. The former preserves the incoming patch representation while injecting explicit local structural evidence, whereas the latter stabilizes long-range state-space evolution over the full token sequence. Consequently, LGAM combines explicit local structural reasoning with efficient global propagation while maintaining the geometry-oriented role of the representation. A self-contained implementation trace, including tensor layouts, classification-token handling, and exact residual ordering, is provided in Appendix A.2.

### 3.4. Saliency-Oriented Evolution with RFCA

**Figure 5:**
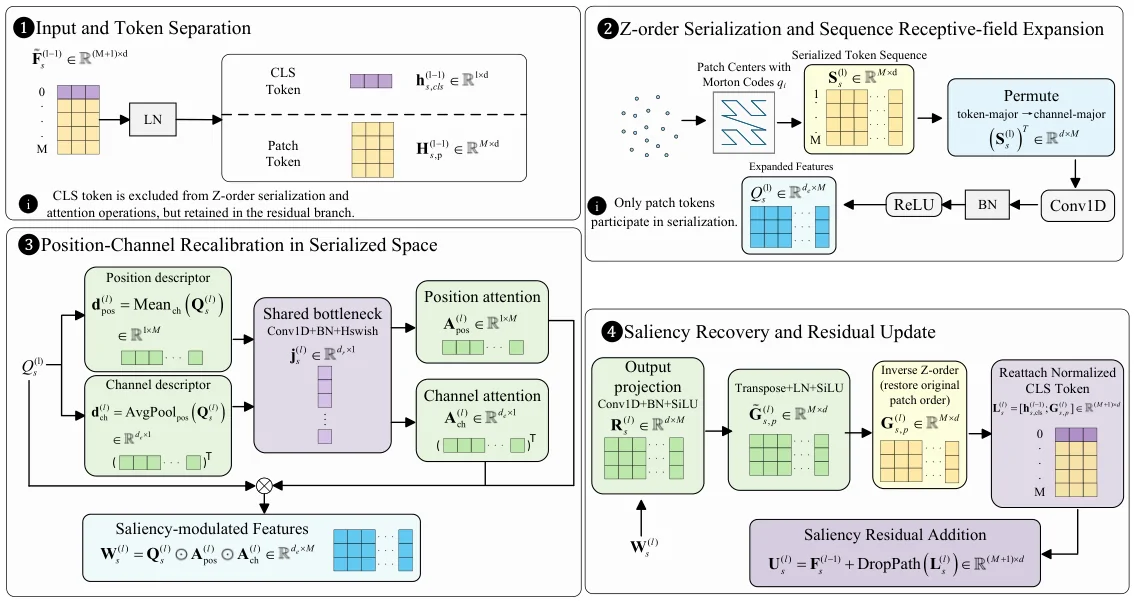
Detailed architecture of the proposed saliency-oriented evolution module based on Receptive-Field Coordinate Attention (RFCA). RFCA first serializes patch tokens according to the Morton Z-order of their patch centers and expands the receptive field using a depthwise one-dimensional convolution. Serialized-position and expanded-channel descriptors are then jointly encoded to generate position-wise and channel-wise attention weights. The recalibrated patch responses are projected to the original embedding dimension, restored to their original patch order through inverse Z-order permutation, and combined with the residual branch.

Receptive-Field Coordinate Attention (RFCA) instantiates the saliency-oriented evolution operator. As illustrated in Figure 5, RFCA adapts receptive-field attention [35] and coordinate-aware channel recalibration [34] to irregular point-cloud patches through a serialization–recalibration–recovery process. Patch tokens are first serialized according to the Morton Z-order of their patch centers. A depthwise one-dimensional convolution then expands the receptive field along the serialized sequence and generates expanded-channel responses. Based on these responses, RFCA jointly estimates serialized-position and expanded-channel attention weights to selectively emphasize task-relevant evidence. Finally, the recalibrated features are projected back to the original embedding dimension and restored to their original patch order through the inverse serialization permutation.

We consider the serialization strategy jointly with LGAM’s role in the dual-stream architecture. Since LGAM already explicitly aggregates Euclidean neighborhoods, RFCA sequence windows need not maximize local-neighborhood preservation again. Instead, they may benefit from retaining spatial structure while incorporating patches outside the direct neighborhoods used by LGAM. We therefore hypothesize that Morton Z-order serialization provides a suitable compromise between local spatial organization and coverage beyond these neighborhoods within the current dual-stream architecture. Section 4.3 examines this interpretation through neighborhood-overlap and window-dispersion measurements, and reports perturbations that probe the frame dependence of the serialization.

At stage *l*, the position-enriched saliency representation is normalized and partitioned as

(20) $$H_{s}^{\left(l-1\right)}={\mathrm{LN}}_{s,1}^{\left(l\right)}\left({\tilde{F}}_{s}^{\left(l-1\right)}\right)=\left[h_{s,\mathrm{cls}}^{\left(l-1\right)};H_{s,p}^{\left(l-1\right)}\right]\in R^{T\times d}.$$

Point-cloud patches have no intrinsic sequential order. RFCA therefore computes coordinate-normalized Morton codes from the patch centers and obtains a Z-order permutation $\Pi_{z}$. The patch tokens are serialized as

(21) $$S_{s}^{\left(l\right)}=\Pi_{z}H_{s,p}^{\left(l-1\right)}\in R^{M\times d}.$$

The classification token is excluded from this permutation because it has no physical patch center. A depthwise one-dimensional convolution with kernel size κ expands the receptive-field response over neighboring positions in the serialized sequence,

(22) $$\begin{array}{cc} Q_{s}^{\left(l\right)}\; & =\mathrm{ReLU}\left({\mathrm{BN}}_{e}^{\left(l\right)}\left({\mathrm{DWConv}}_{\kappa }^{\left(l\right)}\left({\left(S_{s}^{\left(l\right)}\right)}^{T}\right)\right)\right)\in R^{d_{e}\times M},\\ d_{e} & =\kappa d. \end{array}$$

Here, κ specifies both the convolution kernel size and the depthwise channel multiplier in our implementation. Each input channel therefore produces κ output channels, yielding the expanded feature dimension $d_{e}=\kappa d$.

RFCA then summarizes the expanded response along complementary dimensions,

(23) $$\begin{array}{cc} d_{\mathrm{pos}}^{\left(l\right)}\; & ={\mathrm{Mean}}_{\mathrm{ch}}\left(Q_{s}^{\left(l\right)}\right)\in R^{1\times M},\\ d_{\mathrm{ch}}^{\left(l\right)}\; & ={\mathrm{AvgPool}}_{\mathrm{pos}}\left(Q_{s}^{\left(l\right)}\right)\in R^{d_{e}\times 1}. \end{array}$$

The transposed position descriptor and channel descriptor are concatenated and encoded by a shared bottleneck,

(24) $$j_{s}^{\left(l\right)}=\eta^{\left(l\right)}\left({\mathrm{Concat}}_{\mathrm{ch}}\left({\left(d_{\mathrm{pos}}^{\left(l\right)}\right)}^{T},d_{\mathrm{ch}}^{\left(l\right)}\right)\right),$$

where $\eta^{\left(l\right)}$ denotes the shared 1×1 projection followed by BatchNorm and HSwish. Concatenation is performed along the channel dimension, yielding a tensor of size $(M+d_{e})\times 1$, which $\eta^{\left(l\right)}$ maps to $j_{s}^{\left(l\right)}\in R^{d_{r}\times 1}$, where $d_{r}$ is the bottleneck width.

Two independent projections generate the serialized-position and expanded-channel attention maps,

(25) $$\begin{array}{cc} A_{\mathrm{pos}}^{\left(l\right)}\; & ={\left[\sigma \left(\phi_{\mathrm{pos}}^{\left(l\right)}\left(j_{s}^{\left(l\right)}\right)\right)\right]}^{T}\in R^{1\times M},\\ A_{\mathrm{ch}}^{\left(l\right)}\; & =\sigma \left(\phi_{\mathrm{ch}}^{\left(l\right)}\left(j_{s}^{\left(l\right)}\right)\right)\in R^{d_{e}\times 1}. \end{array}$$

where $\phi_{\mathrm{pos}}^{\left(l\right)}$ and $\phi_{\mathrm{ch}}^{\left(l\right)}$ are independent 1×1 projections that map $j_{s}^{\left(l\right)}\in R^{d_{r}\times 1}$ to tensors of size M×1 and $d_{e}\times 1$, respectively. The position response is transposed after sigmoid activation to obtain $A_{\mathrm{pos}}^{\left(l\right)}\in R^{1\times M}$.

The two maps are broadcast over complementary dimensions and jointly recalibrate the expanded receptive-field response,

(26) $$W_{s}^{\left(l\right)}=Q_{s}^{\left(l\right)}⊙A_{\mathrm{pos}}^{\left(l\right)}⊙A_{\mathrm{ch}}^{\left(l\right)}\in R^{d_{e}\times M}.$$

The recalibrated response is first projected from $d_{e}$ to *d* channels,

(27) $$R_{s}^{\left(l\right)}=\mathrm{SiLU}\left({\mathrm{BN}}_{o}^{\left(l\right)}\left(\Phi_{o}^{\left(l\right)}\left(W_{s}^{\left(l\right)}\right)\right)\right)\in R^{d\times M},$$

where $\Phi_{o}^{\left(l\right)}$ is a shared 1×1 Conv1d projection. The implementation then restores the token-major layout, performs output normalization and activation, and finally applies the inverse Z-order permutation,

(28) $$\begin{array}{cc} {\tilde{G}}_{s,p}^{\left(l\right)}\; & =\mathrm{SiLU}\left({\mathrm{LN}}_{s,o}^{\left(l\right)}\left({\left(R_{s}^{\left(l\right)}\right)}^{T}\right)\right)\in R^{M\times d},\\ G_{s,p}^{\left(l\right)}\; & =\Pi_{z}^{-1}{\tilde{G}}_{s,p}^{\left(l\right)}\in R^{M\times d}. \end{array}$$

The recalibrated patch responses are restored to their original patch order before the residual update. The normalized classification token bypasses Z-order serialization, receptive-field expansion, and position–channel recalibration, but is retained in the RFCA residual branch,

(29) $$\begin{array}{cc} L_{s}^{\left(l\right)}\; & =\left[h_{s,\mathrm{cls}}^{\left(l-1\right)};G_{s,p}^{\left(l\right)}\right]\in R^{(M+1)\times d},\\ U_{s}^{\left(l\right)}\; & ={\tilde{F}}_{s}^{\left(l-1\right)}+\mathrm{DropPath}\left(L_{s}^{\left(l\right)}\right)\in R^{(M+1)\times d}. \end{array}$$

LGAM constructs relations directly within Euclidean neighborhoods, whereas RFCA forms receptive fields over serialized positions and uses input-dependent position–channel modulation to recalibrate their responses. These sequence windows can include patches outside the direct LGAM neighborhoods. The two operators thus provide different mechanisms for representation evolution, although their neighborhood coverage may overlap. Further tensor dimensions and implementation details are given in Appendix A.4.

### 3.5. Bidirectional Interaction and Token-Wise Adaptive Gated Fusion

Role-specific evolution encourages the geometry and saliency representations to develop differentiated characteristics. However, maintaining completely isolated streams would prevent each trajectory from exploiting complementary evidence discovered by the other. ORE-Mamba therefore inserts a synchronous bidirectional residual interaction after every pair of LGAM and RFCA blocks, while postponing irreversible integration until the final stage.

Given the role-specific outputs $U_{g}^{\left(l\right)}$ and $U_{s}^{\left(l\right)}$, the two streams are independently normalized and mapped through direction-specific projections,

(30) $$\begin{array}{cccc} {\bar{U}}_{g}^{\left(l\right)}\; & ={\mathrm{LN}}_{i,g}^{\left(l\right)}\left(U_{g}^{\left(l\right)}\right),\; & {\bar{U}}_{s}^{\left(l\right)}\; & ={\mathrm{LN}}_{i,s}^{\left(l\right)}\left(U_{s}^{\left(l\right)}\right),\\ \Delta U_{s\to g}^{\left(l\right)}\; & =P_{s\to g}^{\left(l\right)}\left({\bar{U}}_{s}^{\left(l\right)}\right),\; & \Delta U_{g\to s}^{\left(l\right)}\; & =P_{g\to s}^{\left(l\right)}\left({\bar{U}}_{g}^{\left(l\right)}\right). \end{array}$$

Each direction employs an independently parameterized token-wise projection. The exact MLP structure is specified in Appendix A.5.

The normalized states in Equation (30) are both computed before either stream is updated. The two residual updates are therefore synchronous,

(31) $$F_{g}^{\left(l\right)}=U_{g}^{\left(l\right)}+\Delta U_{s\to g}^{\left(l\right)},\;F_{s}^{\left(l\right)}=U_{s}^{\left(l\right)}+\Delta U_{g\to s}^{\left(l\right)}.$$

The interaction exchanges information between corresponding token positions while preserving two separate stream states. At patch positions, it transfers saliency-aware responses to the geometry stream and geometry-aware responses to the saliency stream. Its application to the CLS position exchanges only the two CLS states and does not aggregate patch information into them. The updated streams serve as the inputs to the next stage.

After the final interaction stage, the same final LayerNorm operator is applied separately to the two streams,

(32) $${\bar{F}}_{g}={\mathrm{LN}}_{f}\left(F_{g}^{\left(L\right)}\right),\;{\bar{F}}_{s}={\mathrm{LN}}_{f}\left(F_{s}^{\left(L\right)}\right).$$

For every token, the two normalized features are concatenated along the channel dimension and processed by a lightweight gating function $\psi_{f}:R^{2d}\to R$. The resulting token-wise scalar gate is

(33) $$G_{f}=\sigma \left(\psi_{f}\left({\mathrm{Concat}}_{\mathrm{ch}}\left({\bar{F}}_{g},{\bar{F}}_{s}\right)\right)\right)\in R^{T\times 1}.$$

The gate is predicted independently for every token, including the classification token, and is broadcast across the *d* feature channels. The pre-normalized fused representation and final output are

(34) $$\begin{array}{cc} F_{\mathrm{fuse}}\; & =G_{f}⊙{\bar{F}}_{g}+\left(1-G_{f}\right)⊙{\bar{F}}_{s}\in R^{T\times d},\\ F_{\mathrm{out}}\; & ={\mathrm{LN}}_{f}\left(F_{\mathrm{fuse}}\right)\in R^{T\times d}. \end{array}$$

where ${\mathrm{LN}}_{f}$ denotes a single LayerNorm module reused for the two final streams and the fused representation, with shared learnable scale $\gamma_{f}$ and bias $\beta_{f}$. Each application computes normalization statistics independently from its own input. This sharing provides a common channel-wise affine calibration before and after fusion while keeping the normalization parameterization compact.

Intermediate interaction maintains two collaborating streams, whereas final fusion produces a unified representation. For classification, global information is obtained by max-plus-mean pooling over the fused patch tokens. The pooled descriptor is concatenated with the final CLS state before prediction, retaining the readout form used in Mamba3D [11]. The CLS component is retained only as part of this architectural convention: it is excluded from patch processing, does not aggregate patch information, and acts as an input-independent learned reference state, as detailed in Appendix A.6. The interaction parameterization and exact classification readout are specified in Appendix A.5 and Appendix A.6, respectively.

### 3.6. Complexity Analysis

We analyze the computational complexity of the dual-stream evolution encoder, excluding the initial point tokenization and task-specific prediction heads. Let *M* denote the number of patch tokens, T=M+1 the total number of tokens including the classification token, *d* the embedding dimension, and *L* the number of evolution stages. We further use $k_{g}$ to denote the LGAM neighborhood size, $d_{m}$ the projected state-space mixing dimension, $n_{s}$ the state dimension, $k_{m}$ the convolution width within each state-space branch, and κ the receptive-field expansion factor of RFCA.

At each stage, LGAM performs patch-center neighborhood construction, local edge transformation, and Bi-SSM propagation. Its leading-order complexity is

(35) $$C_{\mathrm{LGAM}}=O\left(M^{2}+Mk_{g}d^{2}+Tdd_{m}+Td_{m}\left(k_{m}+n_{s}\right)\right),$$

where the $M^{2}$ term arises from pairwise patch-center distance computation in the current KNN implementation. The remaining terms correspond to local edge projection, the input and output projections of the state-space mixer, and branch-wise convolution and selective state propagation, respectively.

RFCA performs Morton serialization, depthwise receptive field expansion, and projection from the expanded dimension $d_{e}=\kappa d$ back to the embedding dimension. Its complexity is

(36) $$C_{\mathrm{RFCA}}=O\left(M\log M+M\kappa^{2}d+M\kappa d^{2}\right),$$

where MlogM corresponds to Morton-code sorting. The two direction-specific interaction MLPs and the final token-wise adaptive gate require

(37) $$C_{\mathrm{inter}}=O\left(Td^{2}\right),\;C_{\mathrm{fusion}}=O\left(Td^{2}\right),$$

respectively.

Since T=M+1=Θ(M) and $d_{m}=\Theta \left(d\right)$, while $k_{g}$, $k_{m}$, $n_{s}$, and κ are fixed architectural constants, the complexity of the current implementation can be summarized as

(38) $$\begin{array}{cc} C_{\mathrm{encoder}}\; & =O\left(L\left[M^{2}+M\log M+Md^{2}\right]+Md^{2}\right)\\ \; & =O\left(L\left[M^{2}+M\log M+Md^{2}\right]\right). \end{array}$$

The final $Md^{2}$ term in the first line corresponds to the adaptive fusion performed once after the last evolution stage and is absorbed into the stage-wise leading order in the second line.

The patch centers remain unchanged across evolution stages. Therefore, the patch-center KNN indices and Morton permutation can, in principle, be precomputed once and reused throughout the encoder. Under this cached implementation, the complexity becomes

(39) $$C_{\mathrm{encoder}}^{\mathrm{cached}}=O\left(M^{2}+M\log M+LMd^{2}\right).$$

Hence, apart from neighborhood construction and serialization, the learned role-specific evolution, cross-stream interaction, and adaptive integration operations scale linearly with the number of patch tokens when the embedding dimension is fixed. Detailed module-level derivations are provided in Appendix A.11.

## 4. Evaluation

This section evaluates ORE-Mamba across object classification, part segmentation, and few-shot recognition. The experiments are designed to examine four aspects of the proposed framework: its recognition performance on synthetic and real-world point clouds, its applicability to dense point-level prediction, the transferability of the learned representations under limited supervision, and the contribution of the major architectural components. We further analyze cross-stream interaction and RFCA serialization strategies to better understand the design choices underlying ORE-Mamba.

### 4.1. Experimental Settings

#### 4.1.1. Datasets

We conduct experiments on three widely adopted point-cloud benchmarks: ModelNet40 [39], ScanObjectNN [40], and ShapeNetPart [41]. ModelNet40 contains 12,311 CAD models from 40 object categories and is used for standard object classification and few-shot recognition. ScanObjectNN contains real-world scanned objects and introduces more challenging factors such as background interference, incomplete geometry, and geometric perturbations. Following prior work, we evaluate three commonly used variants, namely OBJ-BG, OBJ-ONLY, and PB-T50-RS. ShapeNetPart contains 16 object categories with 50 annotated semantic parts and is used to evaluate point-cloud part segmentation.

#### 4.1.2. Evaluation Metrics

For object classification, overall accuracy (OA) is reported. For part segmentation, we report category-wise mean intersection-over-union (${\mathrm{mIoU}}_{C}$) and instance-wise mean intersection-over-union (${\mathrm{mIoU}}_{I}$). For few-shot recognition, mean classification accuracy and standard deviation over multiple evaluation runs are reported. For object classification and part segmentation, the results of both ORE-Mamba and Mamba3D, with and without self-supervised pre-training, are reported as mean ± standard deviation over three independent training runs with different random seeds. The corresponding statistics are provided in Table 2 and Table 3. For few-shot recognition, we report the mean and standard deviation of task-level accuracies over 10 predefined tasks for each way–shot configuration. The task construction, adaptation procedure, and model-selection rule are specified in Appendix A.10.

#### 4.1.3. Implementation Details

ORE-Mamba is implemented in PyTorch 1.13.1 and optimized using its built-in AdamW optimizer. All experiments are conducted on a single NVIDIA RTX 3090 GPU with 24 GB of memory. For the ablation studies, all compared configurations are trained and evaluated under the same experimental settings. Benchmark-specific input point counts, patch configurations, and training hyperparameters are summarized in Appendix A.9. Task-specific readouts and training objectives are described in Appendix A.6.

#### 4.1.4. Pre-Training

We pretrain the complete ORE-Mamba encoder on ShapeNet-55 using a Point-MAE-style masked point-cloud reconstruction strategy [24]. The dataset split follows Point-MAE. Here, the term Point-MAE refers to this pre-training strategy rather than to the pretrained weights released with Point-MAE, while the encoder retains the proposed dual-stream architecture, including LGAM and RFCA. The masking procedure, reconstruction objective, and downstream parameter transfer are detailed in Appendix A.8.

#### 4.1.5. Sampling, Augmentation, and Evaluation Protocol

For all methods trained and evaluated in our experiments (marked with † in Table 2), we use the same dataset-specific input point counts and sampling procedures during training and evaluation. The input contains 2048 points for ScanObjectNN and 1024 points for ModelNet40. Training augmentations are also matched across these methods: rotation is applied to ScanObjectNN, while scaling and translation are used for ModelNet40. All results in Table 2 are reported without test-time voting. In particular, ORE-Mamba and the reproduced Mamba3D baseline are compared using matched input point counts, sampling, and augmentation protocols in both the training-from-scratch and pretrained settings. Literature-reported results are included as reference comparisons; their original training and preprocessing protocols are not assumed to match our reproduced experiments.

#### 4.1.6. Serialization Perturbation Evaluation

We additionally evaluate RFCA under two perturbations of its serialization input on ScanObjectNN OBJ-BG without pre-training: paired shuffling of patch centers and features, and randomization of the coordinate frame from which the Morton codes are computed. Both perturbations are applied during training and evaluation, and each condition is trained from scratch and evaluated over three seeds. Classification OA is reported as mean ± standard deviation over these seeds, and all other training settings are kept unchanged relative to the unperturbed model.

For the input-order condition, patch centers and their corresponding features are jointly shuffled at the RFCA serialization input, and RFCA recomputes the serialization order from the shuffled centers. The outputs are mapped back to their original patch correspondence after RFCA processing. Because the serialization is a deterministic function of the center–feature pairs and the correspondence is restored, this permutation leaves the RFCA computation unchanged, apart from tie-breaking among identical Morton codes. The condition therefore serves as an enumeration control: it checks that the pipeline does not depend on the patch enumeration used by the implementation, and any residual difference from the unperturbed model reflects retraining variability.

For the coordinate-frame condition, the patch-center coordinates used to compute the Morton codes are randomly rotated before the codes are constructed. We use either a rotation about the *z*-axis by an angle drawn uniformly from [0,2π) or a rotation about the mean of the patch centers sampled uniformly from SO(3). Only the copy of the centers used for Morton coding is rotated; the patch features, the geometry stream, and the centers and relative offsets used by LGAM retain the original coordinates. Because the rotated coordinates are subsequently normalized per axis, this is not a rigid rotation of the Morton lattice, but it changes the frame in which the Z-order is evaluated. This confines the perturbation to the coordinate frame of the serialization, so that any change beyond training variability is attributable to that frame rather than to rotated inputs elsewhere in the network. The rotation is resampled at each forward pass and is therefore not fixed across training or evaluation.

### 4.2. Comparison with State of the Art

We compare ORE-Mamba with representative CNN-, Transformer-, and state-space-model-based point-cloud approaches to assess its effectiveness across different point-cloud analysis tasks.

#### 4.2.1. Object Classification

As reported in Table 2, without pre-training, ORE-Mamba achieves a mean OA of 94.0% on ModelNet40, exceeding the three-run mean of Mamba3D by 0.2 percentage points. On ScanObjectNN, ORE-Mamba achieves mean accuracies of 95.24%, 94.03%, and 92.11% on OBJ-BG, OBJ-ONLY, and PB-T50-RS, respectively, exceeding Mamba3D by 2.31, 1.83, and 0.58 percentage points.

With self-supervised pre-training, ORE-Mamba achieves 95.3% on ModelNet40 and 95.83%, 94.87%, and 94.15% on the three ScanObjectNN variants, respectively. These exceed the corresponding Mamba3D means by 0.3, 1.64, 1.28, and 1.05 percentage points. The largest numerical gains occur on OBJ-BG and OBJ-ONLY, whereas the improvements on ModelNet40 are modest. The gain of 0.58 percentage points on PB-T50-RS without pre-training should also be interpreted alongside the standard deviations of 0.61 and 0.50 percentage points for ORE-Mamba and Mamba3D, respectively. These comparisons quantify differences between three-run means without establishing statistical significance.

#### 4.2.2. Part Segmentation

For ShapeNetPart, we use a four-stage encoder with a feature dimension of 384 and extract patch features from the final stage. The segmentation RFCA branch operates on the original patch order without Morton serialization. A category-conditioned decoder combines interpolated patch features, global pooled descriptors, and the ground-truth object category, which is supplied during both training and evaluation. The task-specific configuration and decoder details are provided in Appendix A.7.

Table 3 reports the ShapeNetPart results. Without pre-training, ORE-Mamba achieves 84.4±0.2% in ${\mathrm{mIoU}}_{C}$, exceeding both the Mamba3D mean and the reported Point-MAE result by 0.5 percentage points. Its ${\mathrm{mIoU}}_{I}$ of 85.8±0.1% exceeds the Mamba3D mean of 85.5±0.2% by 0.3 percentage points and matches the best reported value achieved by APES.

With self-supervised pre-training, ORE-Mamba achieves 84.8±0.1% in ${\mathrm{mIoU}}_{C}$ and 86.0±0.1% in ${\mathrm{mIoU}}_{I}$. Compared with Mamba3D, its category-wise mean is 0.1 percentage points lower, while its instance-wise mean is 0.1 percentage points higher. However, its instance-wise mean remains 0.1 percentage points below the best reported result of Point-MAE. Thus, the segmentation results show higher means than Mamba3D without pre-training and competitive performance with pre-training, with small differences in the latter setting.

**Figure 6:**
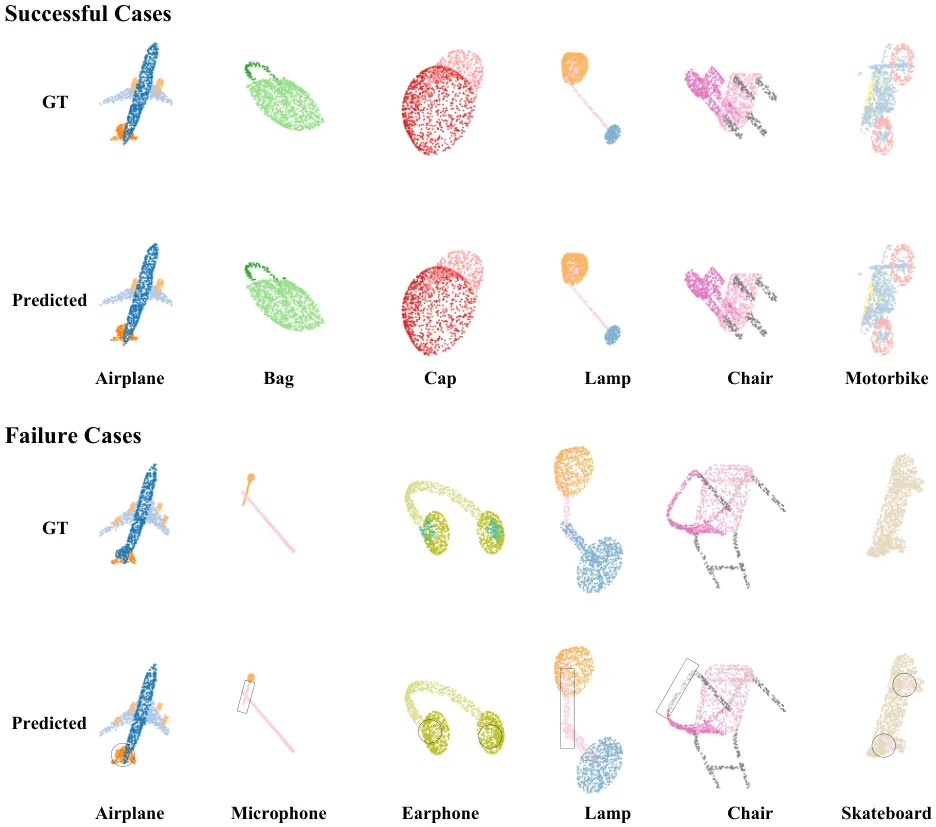
Qualitative part-segmentation results on ShapeNetPart, including successful cases (**top**) and failure cases (**bottom**). Within each group, ground-truth labels (GT) and predictions are shown in the upper and lower rows, respectively. Different colors indicate part labels. Circles and rectangles highlight selected misclassified regions in the failure cases.

Figure 6 presents both successful and failure cases. The successful examples show close agreement between predicted and ground-truth part labels across different object categories. The highlighted failure cases reveal localized errors around thin structures and part junctions, including the airplane tail, lamp stem, and chair supports. These examples illustrate that recovering the overall part layout does not guarantee accurate labeling of small or locally ambiguous regions. Preserving fine-grained part distinctions therefore remains a limitation of the current representation strategy.

#### 4.2.3. Few-Shot Recognition

We evaluate ORE-Mamba under four settings: 5-way 10-shot, 5-way 20-shot, 10-way 10-shot, and 10-way 20-shot. Each setting uses 10 predefined splits. For each *N*-way *K*-shot split, *N* categories are selected, with *K* labeled objects per category from the original training set and 20 objects per category from the original test set. For the reported pretrained results, each split starts from the same ORE-Mamba encoder pretrained on ShapeNet-55 using the Point-MAE strategy, together with a randomly initialized classification head. The encoder and classification head are jointly fine-tuned on the NK labeled training objects. We report mean accuracy and standard deviation across the 10 splits without test-time voting. The training configuration and model-selection rule are detailed in Appendix A.10.

As shown in Table 4, ORE-Mamba achieves 96.6±2.7% and 98.2±1.6% in the two 5-way settings, and 91.9±5.6% and 94.5±3.2% in the two 10-way settings. It achieves the highest reported mean in the 5-way 10-shot setting, but does not consistently outperform the strongest baseline across configurations. These results characterize low-data supervised adaptation under the specified protocol; they do not establish generalization to a fixed, class-disjoint set of novel categories.

#### 4.2.4. Model Complexity and Practical Overhead

Finally, we compare the model complexity of ORE-Mamba with representative point-cloud architectures. As summarized in Table 5, ORE-Mamba contains 15.7 M parameters and requires 4.7 G FLOPs. Its computational cost is not the lowest among existing state-space models; however, it remains within a comparable complexity range while providing strong recognition performance, particularly on the more challenging ScanObjectNN benchmark. These results indicate a favorable trade-off between representational capacity and model complexity.

The objective of ORE-Mamba is to improve point-cloud representations through specialized and collaborative evolution, rather than to minimize inference cost. PointMamba processes serialized point tokens through a plain, non-hierarchical Mamba encoder, while Mamba3D combines Local Norm Pooling with bidirectional state-space modeling. ORE-Mamba maintains separate geometry-oriented and saliency-oriented representations throughout the encoder. In addition to LGAM processing, it performs RFCA-based receptive-field expansion and recalibration, stage-wise bidirectional cross-stream projections, and final token-wise gated fusion. These operations introduce additional computation and intermediate feature storage. Moreover, the current implementation recomputes patch-center KNN indices and Morton permutations at each stage. Indexing, feature reordering, and the associated data movement can affect runtime in ways that parameter counts and FLOPs alone do not fully characterize. Thus, having fewer parameters than Mamba3D does not imply lower inference overhead, and the linear sequence-length scaling of the state-space operations does not guarantee a faster complete model. The recognition results and ablation studies support the effectiveness of the proposed representation design, but its additional overhead remains a limitation for resource-constrained deployment.

### 4.3. Ablation Study

We conduct ablations on ScanObjectNN OBJ-BG without pre-training, covering the architectural components, cross-stream interaction, RFCA serialization and its geometric properties, shared-stream/fusion controls, hyperparameter sensitivity, and role-specific representation analysis (Table 6, Table 7, Table 8, Table 9, Table 10, Table 11 and Table 12). Except for the single-model analyses in Table 9 and Table 12, results are mean ± standard deviation over three independent runs. The perturbation results in Table 10 use models retrained with the corresponding randomization applied during both training and evaluation.

#### 4.3.1. Component Analysis

Table 6 reports the component-wise results. The baseline corresponds to Mamba3D [11] (LNP and Bi-SSM). Replacing LNP with LGA raises OBJ-BG accuracy from 92.93% to 93.73%, and the RFCA-only configuration achieves 93.94%. Adding RFCA to the Mamba3D baseline gives 94.10%, and replacing LNP with LGA in this dual-stream configuration yields the best result, 94.32%. Configurations containing Bi-SSM without LGA retain LNP rather than removing local aggregation entirely. These results support both the LGA replacement and the additional RFCA stream, consistent with their complementary roles.

#### 4.3.2. Cross-Stream Interaction

As shown in Table 7 (top), the model without explicit interaction achieves 94.32% OBJ-BG accuracy. Geometry-to-saliency and saliency-to-geometry interaction reach 94.63% and 94.93%, respectively, and bidirectional interaction yields 95.24%. The two one-way variants gain 0.31 and 0.61 percentage points over no interaction, suggesting that RFCA’s recalibration provides more useful cues to the geometry stream than the reverse, while bidirectional exchange combines both directions.

#### 4.3.3. Shared-Stream Control and Fusion Strategy

To separate persistent dual-stream propagation from model capacity and final fusion, we evaluate a shared-stream control with identical parameterization and three fixed-fusion variants. The control retains every parameterized module (LGAM, RFCA, both interaction projections, gated fusion, and classification head) and changes only the propagated state: after every non-final stage l<L, the two post-interaction outputs are averaged, including the CLS token,

(40) $$X^{\left(l\right)}=\frac{1}{2}\left(F_{g}^{\left(l\right)}+F_{s}^{\left(l\right)}\right),\;l=1,\ldots ,L-1,$$

and both LGAM and RFCA at stage l+1 read this single state after positional re-injection (i.e., $F_{g}^{\left(l\right)}=F_{s}^{\left(l\right)}=X^{\left(l\right)}$ in Equation (8)). The final stage is not averaged and applies the original token-wise gate to the two separate final outputs, so the gate parameters remain active and trained; both interaction projections are also retained. Since the merge adds no parameters, the two configurations instantiate identical parameter shapes and have the same parameter count, and the 15.7 M value in Table 5 is a rounded representation of this same count rather than an equality after rounding.

The fixed-fusion variants replace the final gate by a constant coefficient,

(41) $$F_{\mathrm{fuse}}=\alpha {\bar{F}}_{g}+\left(1-\alpha \right){\bar{F}}_{s},\;\alpha \in \left\{0.5,0.7,0.3\right\},$$

corresponding to LGAM:RFCA ratios of 5:5, 7:3, and 3:7, respectively.

As shown in Table 8, the full model exceeds the shared-stream control by 2.81 percentage points (95.24% versus 92.43%) with the same parameter count and final gate, supporting the benefit of persistent separate states beyond capacity and fusion. Among the fixed-fusion variants, equal weighting is highest (94.15%), and the adaptive gate improves accuracy by 1.09 percentage points; because the fixed variants remove the gate parameters, this gain is not attributable to gate capacity.

#### 4.3.4. RFCA Serialization

Random, Hilbert, and Morton Z-order serialization yield OBJ-BG accuracies of 94.78±0.14%, 94.29±0.23%, and 95.24±0.10% (Table 7, bottom); Morton exceeds Random and Hilbert by 0.46 and 0.95 percentage points. To examine whether this advantage reflects stronger local-neighborhood preservation, we compare the sequence windows induced by each ordering with the Euclidean neighborhoods already aggregated by LGAM.

#### 4.3.5. Neighborhood Overlap and Window Dispersion

Let $N_{g}\left(i\right)$ be the LGAM neighborhood of patch *i* and $W_{s}\left(i\right)$ the other valid patches in its RFCA convolution window under ordering *s* (center and padded positions excluded). The fraction of window neighbors already covered by $N_{g}\left(i\right)$ is

(42) $$R_{s}=\frac{1}{M}\sum_{i=1}^{M}\frac{|W_{s}\left(i\right)\cap N_{g}\left(i\right)|}{|W_{s}\left(i\right)|}.$$

The pairwise dispersion within the window, normalized by the patch-center radius *r*, is

(43) Ds=1M∑i=1M∑j<kj,k∈Ws(i)∥cj−ck∥2|Ws(i)|2r,

where

(44) $$r=\sqrt{\frac{1}{M}\sum_{i=1}^{M}{∥c_{i}-\bar{c}∥}_{2}^{2}},\;\bar{c}=\frac{1}{M}\sum_{i=1}^{M}c_{i},$$

and $c_{i}$ is the center of patch *i*. Normalization by *r* enables comparison across objects of different spatial scales.

As shown in Table 9, Hilbert ordering has higher overlap with LGAM (0.3595 versus 0.3310) and lower within-window dispersion (0.5435 versus 0.6263) than Morton, whereas Random has the lowest overlap (0.0314) and highest dispersion (1.2958); Morton is intermediate in both properties. Together with the accuracy results, this is consistent with the view that maximizing local-neighborhood preservation alone is not optimal when LGAM already performs explicit Euclidean aggregation, and that Morton’s combination of spatial structure and coverage beyond LGAM neighborhoods is a useful compromise. The lower accuracy of Random ordering also indicates that reducing overlap or increasing dispersion alone does not explain performance. These measurements describe window geometry and do not establish feature redundancy, semantic complementarity, or a causal explanation for the accuracy differences; the interpretation is restricted to the evaluated configuration.

#### 4.3.6. Input-Order and Coordinate-Frame Perturbations

Following the protocol in Section 4.1, we perturb the RFCA serialization input. The input-order condition jointly permutes patch centers and features and recomputes the serialization; with correspondence restored, it is an enumeration control rather than a sensitivity test. The rotation conditions randomly rotate only the patch-center coordinates used for Morton coding, about the *z*-axis or about an arbitrary-axis rotation centered at the mean of the patch centers, while patch features and the geometry stream remain unchanged.

As shown in Table 10, paired shuffling yields 95.02±0.21% OA versus 95.24±0.10% unperturbed; the gap of 0.22 percentage points is consistent with the serialization being recomputed from the shuffled centers. With a randomized Morton frame, the *z*-axis and arbitrary-axis conditions yield 94.56±0.19% and 94.81±0.13%, i.e., reductions of 0.68 and 0.43 percentage points; the two rotations differ by 0.25 percentage points. The randomized-frame models retain most of the unperturbed accuracy, but their gap to Hilbert (94.29%) narrows from 0.95 to 0.27 and 0.52 percentage points, and Hilbert was not retrained under the same randomization. These results are consistent with, but do not establish, an ordering-related advantage that persists under frame randomization; they are equally consistent with an advantage partly tied to alignment between the Morton grid and the input frame. The residual reductions also show that serialization is not frame-invariant, so the perturbations qualify, without removing, the spatial-coherence account; a matched randomized-frame comparison with Hilbert and recomputed geometric statistics would test it directly.

These perturbations are not invariance tests and cover OBJ-BG only; the geometric statistics use the input frame only, and whole-cloud rotation is not re-evaluated. Because each condition is retrained and tested under the same randomization, the results reflect accuracy under randomized serialization rather than the sensitivity of a fixed model.

#### 4.3.7. Hyperparameter Sensitivity

Table 11 varies the LGAM neighborhood size $k_{g}\in \left\{2,4,6\right\}$ at κ=7 and the RFCA kernel size κ∈{5,7,9} at $k_{g}=4$. The default (4,7) is best (95.24%). Changing $k_{g}$ to 2 or 6 reduces accuracy by 2.13 or 1.79 percentage points, respectively, whereas changing κ to 5 or 9 reduces it by 1.44 or 2.30 percentage points, respectively. These non-monotonic trends indicate that increasing neighborhood coverage or kernel width alone does not guarantee improved recognition. Because κ also serves as the depthwise channel multiplier ($d_{e}=\kappa d$), this comparison evaluates kernel width and channel expansion jointly.

#### 4.3.8. Role-Specific Representation Analysis

We analyze frozen patch features of the two streams after cross-stream interaction and before final adaptive fusion, using farthest-point sampling and a single forward pass without test-time voting; the CLS token is excluded. The evaluation set contains 581 point clouds from 15 categories, with 128 patch tokens per point cloud. Local geometric preservation is measured by P@4, the overlap between the four nearest neighbors of each patch in patch-center coordinate space ($N_{4}^{x}$) and in $\ell_{2}$-normalized feature space ($N_{4}^{r}$); for a point cloud with *G* patches, the score is

(45) $$P@4_{r}=\frac{1}{G}\sum_{i=1}^{G}\frac{\left|N_{4}^{x}\left(i\right)\cap N_{4}^{r}\left(i\right)\right|}{4},$$

where $N_{4}^{x}\left(i\right)$ and $N_{4}^{r}\left(i\right)$ denote the coordinate- and feature-space neighbor sets and the score is averaged over evaluation point clouds. Category accessibility is measured by the balanced accuracy (mean recall across categories) of a logistic regression probe (C=1.0, L-BFGS, 2000 iterations, feature standardization) fitted separately to the mean-pooled features of each stream on identical training and evaluation partitions, with the evaluation set held out from probe fitting and standardization parameters fitted only on the training features.

As shown in Table 12, LGAM achieves higher P@4 than RFCA (0.3637 versus 0.3327), indicating stronger preservation of local patch-center neighborhoods, whereas RFCA achieves higher probe BA (84.85% versus 81.75%, a difference of approximately 3.1 percentage points), indicating greater linear accessibility of category information under this protocol. Both streams therefore retain category information while exhibiting distinct representational emphases, consistent with the intended bias toward local geometry in LGAM and task-discriminative information in RFCA. The analysis characterizes the final pre-fusion features and does not imply strict functional disentanglement or establish how these differences develop across depth.

#### 4.3.9. Summary

Overall, the experiments demonstrate the effectiveness of ORE-Mamba across object classification, part segmentation, and few-shot recognition, and the component and design analyses show that local geometry aggregation, receptive-field recalibration, and bidirectional cross-stream interaction each contribute to performance. These results support organizing point-cloud representation evolution through complementary role-specific pathways.

## 5. Conclusions

In this work, we revisit point-cloud representation learning from the perspective of representation evolution and introduce objective-driven representation evolution, which organizes feature transformations according to their intended functional roles. Based on this principle, we develop ORE-Mamba, which maintains geometry-oriented and saliency-oriented representations through two distinct yet collaborative evolution pathways.

ORE-Mamba instantiates geometry-oriented evolution through Local Geometry Aggregation Mamba (LGAM), which combines explicit Euclidean neighborhood modeling with long-range Bi-SSM propagation. Saliency-oriented evolution is realized through Receptive-Field Coordinate Attention (RFCA), which couples Morton Z-order serialization with receptive-field-aware position–channel recalibration to concentrate task-discriminative responses. Stage-wise bidirectional residual interaction enables the two role-specific streams to exchange complementary information while remaining distinct across stages, and token-wise adaptive gated fusion integrates their final representations for downstream prediction.

Experiments on ModelNet40, ScanObjectNN, and ShapeNetPart demonstrate strong performance across object classification, part segmentation, and few-shot recognition. The ablation studies further support the contributions of local geometry aggregation, saliency-oriented recalibration, cross-stream interaction, and RFCA serialization. The serialization analysis suggests that the choice of RFCA ordering should be considered jointly with LGAM: Morton exhibits intermediate neighborhood overlap and window dispersion relative to Hilbert and Random, a pattern consistent with its empirical effectiveness in the evaluated dual-stream configuration. These results provide empirical support for organizing heterogeneous functional requirements through distinct yet collaborative representation evolution pathways.

The current ORE-Mamba instantiation considers geometry-oriented and saliency-oriented evolution as a compact two-role design. More generally, the proposed principle could be extended to additional functional roles and adaptive evolution mechanisms according to task requirements, input characteristics, and multimodal information, offering a broader direction for role-aware point-cloud representation learning.

ORE-Mamba may benefit LiDAR-based perception and robotic sensing by combining local geometric modeling with task-relevant feature recalibration. However, its effectiveness on object-centric benchmarks does not establish generalization to large-scale sensing scenes. Non-uniform point density changes the spatial extent of fixed-size neighborhoods, while larger scenes require balancing geometric detail against token count and computational cost. Moreover, the current neighborhood construction incurs an $O(M^{2})$ cost, where *M* is the number of patch tokens. Future work will explore density-adaptive aggregation and scalable tokenization and neighborhood construction, with validation on scene-level sensor data.

Morton serialization remains coordinate-frame dependent. The perturbation experiments randomize, during both training and evaluation, the patch enumeration and the coordinate frame of the centers used only for Morton coding, while patch features and the geometry stream are unchanged; accuracy is largely retained under these randomizations. However, they are not invariance tests, and because Hilbert ordering was not retrained under the same randomization, they do not isolate the ordering effect itself. Moreover, the geometric measurements do not establish a causal explanation for the observed accuracy differences. Further controlled analysis is needed to determine how serialization geometry interacts with the learned representations in the two streams.

## Figures and Tables

**Table 1:** Representation organization in representative point-cloud Mamba methods. Persistent role-specific states are functionally distinct representations propagated separately across stages before final fusion.

Method	Organization	Local Geometry	Serialization	Persistent Role States	Cross-Stream Exchange	Final Output
PointMamba [10]	One non-hierarchical Mamba stream	KNN patches; lightweight PointNet tokenizer	Hilbert and Trans-Hilbert tokens concatenated with order indicators	No; one sequence propagates	No; orders concatenate before encoding	Encoder features used by task heads
Mamba3D [11]	One stream; LNP then bi-SSM per block	KNN-based LNP (K-norm and K-pooling)	Patch-token order with learned positional embeddings; no space-filling curve	No; one state propagates	No; SSM branches merge in each block	Encoder features used by task heads
Pamba [13]	PTv3-derived encoder–decoder with ConvMamba	Sparse submanifold convolutions before global Mamba	Per-block choice of hz or hz-swap (Hilbert/Z-order hybrids)	No separate functional-role states	No; local and global steps are serial	Decoder gives point-wise segmentation
DuoMamba [36]	Two traversal branches per block; one residual state propagates	KNN patches, mini-PointNet, and non-causal Conv1D	Parallel Hilbert and Trans-Hilbert; inverse reorder before summation	No; traversal states merge in each block	No; branches sum and project in-block	Linear layer and average pooling yield a global embedding
ORE-Mamba (Ours)	Geometry and saliency streams persist across stages	LGAM uses patch-center KNN, relative offsets, and Mamba	RFCA uses Morton Z-order; LGAM retains patch-token order	Yes; two role-specific states persist	Yes; bidirectional residual exchange after each stage	Token-wise gate fuses normalized streams for task readout

**Table 2:** Classification results on ModelNet40 and ScanObjectNN. Overall accuracy (%) is reported. ^†^ denotes results obtained through our own training and evaluation; unmarked results are taken from the cited literature. All reported results are without test-time voting. The highest and second-highest values within each training setting are **bolded** and underlined, respectively, using the mean when repeated-run statistics are available. Results for ORE-Mamba and Mamba3D are reported as mean ± standard deviation over three independent training runs in both settings. PT denotes the pre-training strategy. × indicates that self-supervised pre-training was not used; “–” indicates that the result was not reported in the original paper.

Method	ModelNet40	ScanObjectNN			PT
		OBJ-BG	OBJ-ONLY	PB-T50-RS	
*Supervised Learning Only*					
PointNet [3]	89.2	73.3	79.2	68.0	×
PointNet++ [5]	90.7	82.3	84.3	77.9	×
DGCNN [6]	92.2	82.8	86.2	78.1	×
PointMLP [19]	–	–	–	85.42	×
Transformer [42]	91.4	79.86	80.55	77.24	×
Point-MAE ^†^ [24]	92.3	87.43	87.60	81.85	×
PointMamba ^†^ [10]	92.3	90.01	89.67	85.32	×
Mamba3D ^†^ [11]	93.8 ± 0.2	92.93 ± 0.16	92.20 ± 0.26	91.53 ± 0.50	×
SI-Mamba ^†^ [14]	92.3	91.39	91.04	86.78	×
CloudMamba [33]	93.7	–	–	88.3	×
ORE-Mamba (Ours) ^†^	**94.0** ± 0.1	**95.24** ± 0.10	**94.03** ± 0.09	**92.11** ± 0.61	×
*With Self-supervised Pre-training*					
Transformer [42]	92.1	84.85	85.54	78.79	OcCo [43]
Point-BERT ^†^ [23]	93.0	87.60	86.57	83.31	Point-BERT
Point-MAE ^†^ [24]	92.7	89.32	88.64	84.69	Point-MAE
PointMamba ^†^ [10]	92.5	92.42	90.87	87.71	Point-MAE
Mamba3D ^†^ [11]	95.0 ± 0.2	94.19 ± 0.14	93.59 ± 0.19	93.10 ± 0.36	Point-MAE
SI-Mamba ^†^ [14]	92.7	92.94	92.94	88.75	Point-MAE
ORE-Mamba (Ours) ^†^	**95.3** ± 0.2	**95.83** ± 0.08	**94.87** ± 0.09	**94.15** ± 0.14	Point-MAE

**Table 3:** Part segmentation results on ShapeNetPart. Category-wise mean IoU (${\mathrm{mIoU}}_{C}$) and instance-wise mean IoU (${\mathrm{mIoU}}_{I}$) are reported. The highest and second-highest values within each training setting are **bolded** and underlined, respectively, using the mean when repeated-run statistics are available. Results for ORE-Mamba and Mamba3D, both with and without self-supervised pre-training, are reported as mean ± standard deviation over three independent training runs.

Method	${\mathrm{mIoU}}_{C}$ (%)	${\mathrm{mIoU}}_{I}$ (%)
*Supervised Learning Only*		
PointNet [3]	80.4	83.7
PointNet++ [5]	81.9	85.1
DGCNN [6]	82.3	85.2
APES [44]	83.7	**85.8**
Point-MAE [24]	83.9	85.7
PointMamba [10]	83.7	85.7
Mamba3D [11]	83.9 ± 0.2	85.5 ± 0.2
SI-Mamba [14]	82.0	84.9
ORE-Mamba (Ours)	**84.4** ± 0.2	**85.8** ± 0.1
*With Self-supervised Pre-training*		
Point-BERT [23]	83.8	85.6
Point-MAE [24]	84.6	**86.1**
PointMamba [10]	83.8	85.8
Mamba3D [11]	**84.9** ± 0.2	85.9 ± 0.2
SI-Mamba [14]	84.0	86.0
ORE-Mamba (Ours)	84.8 ± 0.1	86.0 ± 0.1

**Table 4:** Few-shot recognition results on ModelNet40. Mean accuracy (%) and standard deviation (%) are reported. For ORE-Mamba, each configuration uses 10 predefined tasks, with 20 query objects per selected category. The reported statistics are computed over the best query-set accuracy obtained during adaptation for each task, without test-time voting. The complete protocol is described in Appendix A.10. Best results are **bolded**, and second-best results are underlined.

Method	5-Way		10-Way	
	10-Shot	20-Shot	10-Shot	20-Shot
*Supervised Learning Only*				
PointNet [3]	52.0 ± 3.8	57.8 ± 4.9	46.6 ± 4.3	35.2 ± 4.8
DGCNN [6]	31.6 ± 2.8	40.8 ± 4.6	19.9 ± 2.1	16.9 ± 1.5
*With Self-supervised Pre-training*				
Point-BERT [23]	94.6 ± 3.1	96.3 ± 2.7	91.0 ± 5.4	92.7 ± 5.1
MaskPoint [45]	95.0 ± 3.7	97.2 ± 1.7	91.4 ± 4.0	93.4 ± 3.5
Point-MAE [24]	96.3 ± 2.5	97.8 ± 1.8	**92.6** ± 4.1	95.0 ± 3.0
PointMamba [10]	95.0 ± 2.3	97.3 ± 1.8	91.4 ± 4.4	92.8 ± 4.0
Mamba3D [11]	96.4 ± 2.2	98.2 ± 1.2	92.4 ± 4.1	**95.2** ± 2.9
SI-Mamba [14]	96.4 ± 2.7	**98.5** ± 1.5	92.0 ± 5.1	95.1 ± 3.6
ORE-Mamba (Ours)	**96.6** ± 2.7	98.2 ± 1.6	91.9 ± 5.6	94.5 ± 3.2

**Table 5:** Comparison of model complexity in terms of parameter count and FLOPs.

Category	Method	Venue	Params (M)	FLOPs (G)
CNN-based	PointNet [3]	CVPR 2017	3.5	0.5
	PointNet++ [5]	NeurIPS 2017	1.5	1.7
	DGCNN [6]	TOG 2019	1.8	2.4
	PointMLP [19]	ICLR 2022	13.2	31.4
Transformer-based	Transformer [42]	NeurIPS 2017	22.1	4.8
	Point-MAE [24]	ECCV 2022	22.1	4.8
	Point-BERT [23]	CVPR 2022	22.1	4.8
State Space Models	PointMamba [10]	NeurIPS 2024	12.3	3.1
	Mamba3D [11]	ACM MM 2024	16.9	3.9
	SI-Mamba [14]	CVPR 2025	12.3	3.6
	CloudMamba [33]	AAAI 2026	10.0	1.2
	ORE-Mamba (Ours)	–	15.7	4.7

**Table 6:** Component-wise ablation study on ScanObjectNN OBJ-BG. Values are reported as mean ± standard deviation over three independent training runs with different random seeds. The check mark (🗸) indicates that the corresponding component is included in the configuration, and “–” indicates that it is not included. Bold indicates the best result.

Local Aggregation	Bi-SSM	RFCA	OBJ-BG (%)
LNP	🗸	–	92.93 ± 0.16
LGA	🗸	–	93.73 ± 0.14
–	–	🗸	93.94 ± 0.07
LNP	🗸	🗸	94.10 ± 0.11
LGA	🗸	🗸	**94.32** ± 0.19

**Table 7:** Design analysis of cross-stream interaction and RFCA serialization strategies on ScanObjectNN OBJ-BG. Values are reported as mean ± standard deviation over three independent training runs with different random seeds. Bold indicates the best result.

Analysis	Variant	OBJ-BG (%)
Interaction	No interaction	94.32 ± 0.19
	Geometry → Saliency	94.63 ± 0.08
	Saliency → Geometry	94.93 ± 0.12
	Bidirectional	**95.24** ± 0.10
Serialization	Random	94.78 ± 0.14
	Hilbert	94.29 ± 0.23
	Z-order	**95.24** ± 0.10

**Table 8:** Shared-stream and fusion-strategy comparisons on ScanObjectNN OBJ-BG without pre-training. All values are reported as mean ± standard deviation over three independent training runs with different random seeds, obtained under the same training settings. The shared-stream control retains the full model’s LGAM blocks, RFCA blocks, interaction projections, gated fusion, and classification head, and changes only the inter-stage state propagation: after each non-final stage, the two post-interaction outputs are averaged into one state (Equation (40)), which both operators read at the next stage. Because the merge adds no parameters, the control and the full model have identical parameter counts. Fixed ratios specify LGAM:RFCA contributions and replace the adaptive gate by a constant coefficient. Bold indicates the best result.

State Propagation	Final Fusion	OA (%)
Shared stream	Adaptive	92.43 ± 0.36
Persistent dual stream	Fixed 5:5	94.15 ± 0.12
Persistent dual stream	Fixed 7:3	93.11 ± 0.25
Persistent dual stream	Fixed 3:7	92.35 ± 0.16
Persistent dual stream	Adaptive	**95.24** ± 0.10

**Table 9:** Neighborhood overlap with LGAM and normalized spatial dispersion of RFCA sequence windows for different serialization strategies on ScanObjectNN OBJ-BG. $R_{s}$ and $D_{s}$ are defined in Equations (42) and (43), respectively.

Ordering	Neighborhood Overlap $R_{s}$	Window Dispersion $D_{s}$
Morton (Z-order)	0.3310	0.6263
Hilbert	0.3595	0.5435
Random	0.0314	1.2958

**Table 10:** RFCA serialization perturbations on ScanObjectNN OBJ-BG. OA is reported as mean ± standard deviation over three independent training seeds, and mean OA reductions are measured in percentage points relative to the unperturbed condition. In the input-order condition, patch centers and features are jointly shuffled at the RFCA serialization input and the serialization is recomputed, which leaves the RFCA computation unchanged apart from tie-breaking; this condition is therefore an enumeration control. In the rotation conditions, only the patch centers used to compute the RFCA Morton codes are randomly rotated; patch features and the geometry stream retain the original coordinates. Each perturbation is applied during both training and evaluation, and each condition is trained from scratch. Bold indicates the best result.

Condition	OA (%)	OA Reduction (pp)
Unperturbed	95.24±0.10	—
Paired shuffling at the RFCA input (control)	95.02±0.21	0.22
Randomized Morton frame, *z*-axis	94.56±0.19	0.68
Randomized Morton frame, arbitrary axis	94.81±0.13	0.43

**Table 11:** Hyperparameter sensitivity on ScanObjectNN OBJ-BG without pre-training. Each result is reported as mean ± standard deviation over three independent training runs with different random seeds. One hyperparameter is varied at a time around the default configuration $\left(k_{g},\kappa \right)=\left(4,7\right)$, while all other experimental settings remain unchanged. Bold indicates the best result.

LGAM Neighborhood Size $k_{g}$	RFCA Kernel Size κ	OA (%)
4	7	**95.24** ± 0.10
2	7	93.11 ± 0.17
6	7	93.45 ± 0.21
4	5	93.80 ± 0.18
4	9	92.94 ± 0.34

**Table 12:** Representation analysis of the two streams immediately before final adaptive fusion. P@4 measures local geometric neighborhood preservation, and BA denotes linear-probe balanced accuracy. Both streams are evaluated on the same 581 point clouds using identical probe training and evaluation partitions. Bold values indicate the higher score for each metric.

Feature Stream	P@4	BA (%)
Geometry-oriented (LGAM)	**0.3637**	81.75
Saliency-oriented (RFCA)	0.3327	**84.85**

## Data Availability

The original contributions presented in the study are included in the article; further inquiries can be directed to the corresponding author.

## Appendix A. Implementation-Aligned Formulation of ORE-Mamba

This appendix complements Section 3 with detailed architectural configurations, tensor layouts, and execution procedures required for implementation. Unless otherwise specified, the batch dimension is omitted for compact notation and retained explicitly when tensor layout is implementation-critical.

### Appendix A.1. Architectural Configuration and Tokenization Details

This subsection specifies the tokenization procedure and the concrete architectural configuration used in the reported experiments. The principal configuration is summarized in Table A1.

**Table A1:** Principal architectural configuration of ORE-Mamba.

Configuration	Value
Number of patches *M*	128
Points per patch $K_{p}$	32
Token dimension *d*	384
Number of evolution stages *L*	4
LGAM neighborhood size $k_{g}$	4
RFCA kernel/channel multiplier κ	7
RFCA reduction ratio	32
RFCA bottleneck dimension $d_{r}$	12
Mamba expansion ratio	2
Mamba inner dimension $d_{m}$	768
Mamba state dimension $n_{s}$	16
Mamba convolution width $k_{m}$	4
Mamba branch type	v4
Cross-stream projection	d→d→d
Gate predictor	2d→d→1

Given an input point cloud $P={\left\{p_{n}\right\}}_{n=1}^{N}\in R^{N\times 3}$, farthest-point sampling selects *M* centers $C={\left[c_{1},\ldots ,c_{M}\right]}^{T}\in R^{M\times 3}$. For every center, its $K_{p}$ nearest input points form a centered patch,

(A1) $$\Delta G_{i}=\left\{p_{j}-c_{i}\;|\;j\in \mathrm{KNN}\left(c_{i},P,K_{p}\right)\right\}\in R^{K_{p}\times 3}.$$

A shared PointNet-style encoder maps every patch to a token,

(A2) $$x_{i}=E_{\mathrm{patch}}\left(\Delta G_{i}\right),X_{p}={\mathrm{Stack}}_{i=1}^{M}\left(x_{i}\right)\in R^{M\times d}.$$

The patch encoder contains two point-wise convolutional stages with symmetric maximum aggregation. Its channel configuration is 3→128→256, followed by concatenation of the point-wise response with its pooled context and a second stage 512→512→d. The positional encoder is an MLP 3→128→d with SiLU between its two linear layers. A learnable classification token $x_{\mathrm{cls}}\in R^{d}$ is prepended to the patch tokens. The positional sequence consists of a learnable classification-token positional embedding $z_{\mathrm{cls}}\in R^{d}$ and an MLP embedding of the patch centers,

(A3) $$\begin{array}{cc} X^{\left(0\right)}\; & =\left[x_{\mathrm{cls}};X_{p}\right]\in R^{T\times d},\\ Z\; & =\left[z_{\mathrm{cls}};E_{\mathrm{pos}}\left(C\right)\right]\in R^{T\times d},\;T=M+1=129. \end{array}$$

Both streams are initialized from the same token sequence,

(A4) $$F_{g}^{\left(0\right)}=F_{s}^{\left(0\right)}=X^{\left(0\right)},$$

and positional information is re-injected before every role-specific stage,

(A5) $${\tilde{F}}_{g}^{\left(l-1\right)}=F_{g}^{\left(l-1\right)}+Z,\;{\tilde{F}}_{s}^{\left(l-1\right)}=F_{s}^{\left(l-1\right)}+Z.$$

### Appendix A.2. Implementation Details of LGAM

Section 3.3 introduces LGAM at the mechanism level. To keep the implementation trace continuous, this subsection restates its normalized input, local relation update, and final residual output, while adding the batched tensor layouts and execution order abstracted in the main text. Its normalized input is

(A6) $$H_{g}^{\left(l-1\right)}={\mathrm{LN}}_{g,1}^{\left(l\right)}\left({\tilde{F}}_{g}^{\left(l-1\right)}\right)=\left[h_{g,\mathrm{cls}}^{\left(l-1\right)};H_{g,p}^{\left(l-1\right)}\right]\in R^{T\times d}.$$

The classification token has no physical patch center and is excluded from Euclidean neighborhood construction. In the batched implementation, $C\in R^{B\times M\times 3}$ is used to form the pairwise center-distance matrix. For each center, the implementation retrieves the $k_{g}+1$ smallest distances and removes the first index corresponding to the center itself,

(A7) $$N_{k_{g}}\left(i\right)={\mathrm{TopK}}_{k_{g}+1}^{\mathrm{smallest}}\left({\left\{{∥c_{i}-c_{j}∥}_{2}\right\}}_{j=1}^{M}\right)\setminus \left\{i\right\}.$$

The neighborhood indices depend only on the patch centers and can therefore be cached across evolution stages, although the current implementation recomputes them inside each LGAM block. For every $j\in N_{k_{g}}\left(i\right)$, LGAM constructs

(A8) $$e_{ij}^{\left(l\right)}={\mathrm{Concat}}_{\mathrm{ch}}\left(h_{g,i}^{\left(l-1\right)},h_{g,j}^{\left(l-1\right)}-h_{g,i}^{\left(l-1\right)},c_{j}-c_{i}\right)\in R^{2d+3}.$$

For batched execution, these relations are stacked in the channel-major layout

(A9) $$E_{g}^{\left(l\right)}\in R^{B\times \left(2d+3\right)\times M\times k_{g}}.$$

A shared 1×1 edge projection, implemented by Conv2d, transforms every center–neighbor relation independently,

(A10) $$z_{ij}^{\left(l\right)}=\phi_{e}^{\left(l\right)}\left(e_{ij}^{\left(l\right)}\right)=e_{ij}^{\left(l\right)}W_{e}^{\left(l\right)}+b_{e}^{\left(l\right)}\in R^{2d},$$

where $W_{e}^{\left(l\right)}\in R^{(2d+3)\times 2d}$ and $b_{e}^{\left(l\right)}\in R^{2d}$. Channel-wise maximum aggregation over the $k_{g}$ neighbors gives

(A11) $$m_{g,i}^{\left(l\right)}=\max_{j\in N_{k_{g}}\left(i\right)}z_{ij}^{\left(l\right)}\in R^{2d}.$$

After Conv2d, the edge tensor has layout $B\times 2d\times M\times k_{g}$, and maximum aggregation is performed over the last (neighbor) dimension. The resulting tensor $M_{g,\mathrm{ch}}^{\left(l\right)}\in R^{B\times 2d\times M}$ is permuted to B×M×2d for LayerNorm, returned to channel-major layout for the 1×1 Conv1d projection, and then restored to token-major layout. At the token level, the corresponding operation is

(A12) $$g_{g,i}^{\left(l\right)}=\mathrm{SiLU}\left({\mathrm{LN}}_{e}^{\left(l\right)}\left(m_{g,i}^{\left(l\right)}\right)W_{g}^{\left(l\right)}+b_{g}^{\left(l\right)}\right)\in R^{d},$$

where $W_{g}^{\left(l\right)}\in R^{2d\times d}$ and $b_{g}^{\left(l\right)}\in R^{d}$. The exact local-branch execution order is

(A13) $$\mathrm{Conv2d}\to {\mathrm{MaxPool}}_{\mathrm{neighbor}}\to \mathrm{LayerNorm}\to \mathrm{Conv1d}\to \mathrm{SiLU}.$$

Stacking all patch responses yields

(A14) $$G_{g,p}^{\left(l\right)}={\mathrm{Stack}}_{i=1}^{M}\left(g_{g,i}^{\left(l\right)}\right)\in R^{M\times d}.$$

Consistent with the implementation, the normalized classification token is forwarded through the local branch without participating in neighborhood aggregation. The resulting local residual update is

(A15) $$\begin{array}{cc} L_{g}^{\left(l\right)}\; & =\left[h_{g,\mathrm{cls}}^{\left(l-1\right)};G_{g,p}^{\left(l\right)}\right]\in R^{T\times d},\\ {\hat{F}}_{g}^{\left(l\right)}\; & ={\tilde{F}}_{g}^{\left(l-1\right)}+\mathrm{DropPath}\left(L_{g}^{\left(l\right)}\right). \end{array}$$

The local residual is completed before long-range state-space evolution. The locally updated representation is first normalized,

(A16) $$H_{m}^{\left(l\right)}={\mathrm{LN}}_{g,2}^{\left(l\right)}\left({\hat{F}}_{g}^{\left(l\right)}\right)\in R^{T\times d},$$

and is then processed by the Bi-SSM $B^{\left(l\right)}$ detailed in Appendix A.3. The final LGAM output is

(A17) $$U_{g}^{\left(l\right)}={\hat{F}}_{g}^{\left(l\right)}+\mathrm{DropPath}\left(B^{\left(l\right)}\left(H_{m}^{\left(l\right)}\right)\right)\in R^{T\times d}.$$

Thus, the exact residual ordering is

(A18) $${\tilde{F}}_{g}^{\left(l-1\right)}\overset{{\mathrm{LN}}_{g,1}^{\left(l\right)}+\mathrm{local}\;\mathrm{aggregation}}{\to }{\hat{F}}_{g}^{\left(l\right)}\overset{{\mathrm{LN}}_{g,2}^{\left(l\right)}+B^{\left(l\right)}}{\to }U_{g}^{\left(l\right)}.$$

The two residual sublayers are therefore serial rather than parallel: the state-space mixer evolves the locally enriched representation, not the original stage input.

### Appendix A.3. Projected-Channel Bidirectional SSM

This subsection specifies the internal dimensions, channel indexing, and parameterization of the mixer $B^{\left(l\right)}$.

With expansion ratio 2, the inner Mamba dimension is $d_{m}=2d=768$. The shared input projection maps the normalized token state to a joint content–gate representation of dimension $2d_{m}=1536$,

(A19) $$V^{\left(l\right)}=P_{\mathrm{in}}^{\left(l\right)}\left(H_{m}^{\left(l\right)}\right)\in R^{T\times 2d_{m}}.$$

Let $M_{+}^{\left(l\right)}$ and $M_{-}^{\left(l\right)}$ denote the complete branch computations, including content–gate decomposition, branch-specific depthwise convolution, selective state-space propagation, and multiplicative gating. The two branch responses are

(A20) $$\begin{array}{cc} Y_{+}^{\left(l\right)}\; & =M_{+}^{\left(l\right)}\left(V^{\left(l\right)}\right),\\ Y_{-}^{\left(l\right)}\; & =R_{c}^{-1}\left[M_{-}^{\left(l\right)}\left(R_{c}\left(V^{\left(l\right)}\right)\right)\right], \end{array}\;Y_{+}^{\left(l\right)},Y_{-}^{\left(l\right)}\in R^{T\times d_{m}}.$$

For a token-major tensor $A\in R^{T\times D}$, the reversal operator is defined by

(A21) $${\left[R_{c}\left(A\right)\right]}_{t,c}=A_{t,D+1-c}.$$

Here, $D=2d_{m}$ at the branch input and $D=d_{m}$ at the branch output. Both branches use causal depthwise convolution and selective scanning along the token axis. Thus, within the mixer,

(A22) $$\frac{\partial Y_{\pm ,t}^{\left(l\right)}}{\partial V_{u}^{\left(l\right)}}=0,\;u>t.$$

The branches share the input and output projections but have independent convolutional and state-space parameters. Their responses are summed and projected back to the token embedding dimension,

(A23) $$B^{\left(l\right)}\left(H_{m}^{\left(l\right)}\right)=P_{\mathrm{out}}^{\left(l\right)}\left(Y_{+}^{\left(l\right)}+Y_{-}^{\left(l\right)}\right)\in R^{T\times d}.$$

Together with Equation (A17), this closes the complete local-to-global LGAM computation.

### Appendix A.4. Implementation Details of RFCA

Section 3.4 defines RFCA at the mechanism level. This subsection repeats its input–recalibration–output skeleton so that the exact Morton-code construction, tensor layouts, and recovery order can be followed without repeatedly returning to the main text. Its normalized input is

(A24) $$H_{s}^{\left(l-1\right)}={\mathrm{LN}}_{s,1}^{\left(l\right)}\left({\tilde{F}}_{s}^{\left(l-1\right)}\right)=\left[h_{s,\mathrm{cls}}^{\left(l-1\right)};H_{s,p}^{\left(l-1\right)}\right]\in R^{T\times d}.$$

For each sample, the patch-center coordinates are independently normalized along the three axes and quantized to 21-bit integers,

(A25) $${\hat{c}}_{i}=\left\lfloor \frac{c_{i}-c_{\min}}{c_{\max}-c_{\min}+\epsilon }\left(2^{21}-1\right)\right\rfloor .$$

Interleaving the bits of the quantized *x*-, *y*-, and *z*-coordinates gives the Morton code. Sorting these codes induces the Z-order permutation $\Pi_{z}$, and sorting the permutation indices gives $\Pi_{z}^{-1}$. The indices depend only on C and may be cached across stages, although the current implementation recomputes them in each RFCA block. The classification token is excluded because it has no physical patch center. The serialized patch representation is

(A26) $$S_{s}^{\left(l\right)}=\Pi_{z}H_{s,p}^{\left(l-1\right)}\in R^{M\times d}.$$

With the batch dimension retained, serialization preserves token-major layout, B×M×d, before a transpose produces the channel-major Conv1d layout B×d×M.

A depthwise one-dimensional convolution with kernel size κ and channel multiplier κ expands the serialized response to $d_{e}=\kappa d=2688$ channels,

(A27) $$Q_{s}^{\left(l\right)}=\mathrm{ReLU}\left({\mathrm{BN}}_{e}^{\left(l\right)}\left({\mathrm{DWConv}}_{\kappa }^{\left(l\right)}\left({\left(S_{s}^{\left(l\right)}\right)}^{T}\right)\right)\right)\in R^{d_{e}\times M}.$$

In batched form, $Q_{s}^{\left(l\right)}\in R^{B\times d_{e}\times M}$. The operation is depthwise with respect to the *d* input channels but does not preserve their number: each input channel produces κ receptive-field responses.

RFCA constructs complementary descriptors by averaging over expanded channels and serialized positions,

(A28) $$\begin{array}{cc} d_{\mathrm{pos}}^{\left(l\right)}\; & ={\mathrm{Mean}}_{\mathrm{ch}}\left(Q_{s}^{\left(l\right)}\right)\in R^{1\times M},\\ d_{\mathrm{ch}}^{\left(l\right)}\; & ={\mathrm{AvgPool}}_{\mathrm{pos}}\left(Q_{s}^{\left(l\right)}\right)\in R^{d_{e}\times 1}. \end{array}$$

The position descriptor is transposed and concatenated with the channel descriptor along the Conv1d channel dimension. A shared bottleneck of dimension

(A29) $$d_{r}=\max\left(8,\left\lfloor \frac{d}{32}\right\rfloor \right)=12$$

produces the joint context vector,

(A30) $$\begin{array}{cc} q_{s}^{\left(l\right)}\; & ={\mathrm{Concat}}_{\mathrm{ch}}\left({\left(d_{\mathrm{pos}}^{\left(l\right)}\right)}^{T},d_{\mathrm{ch}}^{\left(l\right)}\right)\in R^{(M+\kappa d)\times 1},\\ j_{s}^{\left(l\right)}\; & =\mathrm{HSwish}\left({\mathrm{BN}}_{r}^{\left(l\right)}\left({\mathrm{Conv}}_{1\times 1,r}^{\left(l\right)}\left(q_{s}^{\left(l\right)}\right)\right)\right)\in R^{d_{r}\times 1}. \end{array}$$

Two independent 1×1 projections generate the serialized-position and expanded-channel attention maps,

(A31) $$\begin{array}{cc} A_{\mathrm{pos}}^{\left(l\right)}\; & ={\left[\sigma \left({\mathrm{Conv}}_{1\times 1,\mathrm{pos}}^{\left(l\right)}\left(j_{s}^{\left(l\right)}\right)\right)\right]}^{T}\in R^{1\times M},\\ A_{\mathrm{ch}}^{\left(l\right)}\; & =\sigma \left({\mathrm{Conv}}_{1\times 1,\mathrm{ch}}^{\left(l\right)}\left(j_{s}^{\left(l\right)}\right)\right)\in R^{d_{e}\times 1}. \end{array}$$

Broadcast multiplication recalibrates the expanded response,

(A32) $$W_{s}^{\left(l\right)}=Q_{s}^{\left(l\right)}⊙A_{\mathrm{pos}}^{\left(l\right)}⊙A_{\mathrm{ch}}^{\left(l\right)}\in R^{d_{e}\times M}.$$

The recalibrated response is projected from $d_{e}$ to *d* channels by a 1×1 Conv1d followed by BatchNorm and SiLU,

(A33) $$R_{s}^{\left(l\right)}=\mathrm{SiLU}\left({\mathrm{BN}}_{o}^{\left(l\right)}\left({\mathrm{Conv}}_{1\times 1,o}^{\left(l\right)}\left(W_{s}^{\left(l\right)}\right)\right)\right)\in R^{d\times M}.$$

The implementation then restores the token-major layout, applies LayerNorm and SiLU, and finally applies the inverse Z-order permutation,

(A34) $$\begin{array}{cc} {\tilde{G}}_{s,p}^{\left(l\right)}\; & =\mathrm{SiLU}\left({\mathrm{LN}}_{s,o}^{\left(l\right)}\left({\left(R_{s}^{\left(l\right)}\right)}^{T}\right)\right)\in R^{M\times d},\\ G_{s,p}^{\left(l\right)}\; & =\Pi_{z}^{-1}{\tilde{G}}_{s,p}^{\left(l\right)}\in R^{M\times d}. \end{array}$$

The inverse permutation is applied only after the response has returned to token-major layout and undergone token-wise post-processing. The classification token is excluded from Z-order serialization, receptive-field expansion, and position–channel recalibration, but its normalized representation is forwarded through the RFCA residual branch,

(A35) $$\begin{array}{cc} L_{s}^{\left(l\right)}\; & =\left[h_{s,\mathrm{cls}}^{\left(l-1\right)};G_{s,p}^{\left(l\right)}\right]\in R^{T\times d},\\ U_{s}^{\left(l\right)}\; & ={\tilde{F}}_{s}^{\left(l-1\right)}+\mathrm{DropPath}\left(L_{s}^{\left(l\right)}\right)\in R^{T\times d}. \end{array}$$

### Appendix A.5. Interaction and Token-Wise Adaptive Gated Fusion

To preserve a continuous implementation trace, this subsection restates the interaction update and final integration equations from Section 3.5, then specifies their MLP parameterization, synchrony, token coverage, and normalization sharing. After role-specific evolution, the two streams exchange information through separate cross-stream projections,

(A36) $$\begin{array}{cccc} {\bar{U}}_{g}^{\left(l\right)}\; & ={\mathrm{LN}}_{i,g}^{\left(l\right)}\left(U_{g}^{\left(l\right)}\right),\; & {\bar{U}}_{s}^{\left(l\right)}\; & ={\mathrm{LN}}_{i,s}^{\left(l\right)}\left(U_{s}^{\left(l\right)}\right),\\ \Delta U_{s\to g}^{\left(l\right)}\; & =P_{s\to g}^{\left(l\right)}\left({\bar{U}}_{s}^{\left(l\right)}\right),\; & \Delta U_{g\to s}^{\left(l\right)}\; & =P_{g\to s}^{\left(l\right)}\left({\bar{U}}_{g}^{\left(l\right)}\right),\\ F_{g}^{\left(l\right)}\; & =U_{g}^{\left(l\right)}+\Delta U_{s\to g}^{\left(l\right)},\; & F_{s}^{\left(l\right)}\; & =U_{s}^{\left(l\right)}+\Delta U_{g\to s}^{\left(l\right)}. \end{array}$$

Each cross-stream projection is a two-layer MLP,

(A37) $$P^{\left(l\right)}\left(X\right)=\left[\mathrm{GELU}\left(XW_{1}^{\left(l\right)}+b_{1}^{\left(l\right)}\right)\right]W_{2}^{\left(l\right)}+b_{2}^{\left(l\right)},$$

Both linear layers map d→d. Parameters are specific to each stage and interaction direction and are shared across token positions. For every t=1,…,T, the projection is applied independently to the feature vector at position *t*; the CLS token occupies t=1. Both normalized inputs and interaction increments are computed before either residual update, ensuring synchronous updates.

After the final stage, a single parameter-shared LayerNorm module is invoked separately for the two streams,

(A38) $${\bar{F}}_{g}={\mathrm{LN}}_{f}\left(F_{g}^{\left(L\right)}\right),\;{\bar{F}}_{s}={\mathrm{LN}}_{f}\left(F_{s}^{\left(L\right)}\right).$$

Their channel-wise concatenation is processed by $\psi_{f}:R^{2d}\to R$, implemented token-wise as Linear(2d,d)–GELU–Linear(d,1),

(A39) $$G_{f}=\sigma \left(\psi_{f}\left({\mathrm{Concat}}_{\mathrm{ch}}\left({\bar{F}}_{g},{\bar{F}}_{s}\right)\right)\right)\in R^{T\times 1}.$$

The scalar gate is predicted independently for every token, including the classification token, and is broadcast over feature channels; it is therefore a token-wise rather than channel-wise gate. The fused representation is post-normalized by the same final LayerNorm module,

(A40) $$\begin{array}{cc} F_{\mathrm{fuse}}\; & =G_{f}⊙{\bar{F}}_{g}+\left(1-G_{f}\right)⊙{\bar{F}}_{s}\in R^{T\times d},\\ F_{\mathrm{out}}\; & ={\mathrm{LN}}_{f}\left(F_{\mathrm{fuse}}\right)\in R^{T\times d}. \end{array}$$

The same affine parameters of ${\mathrm{LN}}_{f}$ are therefore reused for three calls: normalization of $F_{g}^{\left(L\right)}$, normalization of $F_{s}^{\left(L\right)}$, and post-normalization of $F_{\mathrm{fuse}}$. Each call nevertheless computes normalization statistics from its own input tensor.

### Appendix A.6. Task-Specific Readouts and Training Objectives

Let $F_{\mathrm{out},p}\in R^{M\times d}$ contain the final patch features and $F_{\mathrm{out},\mathrm{cls}}\in R^{d}$ denote the first-token state. The classification readout is

(A41) $$\begin{array}{cc} p_{\mathrm{global}}\; & =\mathrm{MaxPool}\left(F_{\mathrm{out},p}\right)+\mathrm{MeanPool}\left(F_{\mathrm{out},p}\right)\in R^{d},\\ f_{\mathrm{read}}\; & ={\mathrm{Concat}}_{\mathrm{ch}}\left(F_{\mathrm{out},\mathrm{cls}},p_{\mathrm{global}}\right)\in R^{2d},\\ \hat{y}\; & =H_{\mathrm{cls}}\left(f_{\mathrm{read}}\right). \end{array}$$

MaxPool and MeanPool operate over the *M* patch positions independently for each channel, excluding the CLS token; their sum has dimension *d*, and the concatenated classifier input has dimension 2d.

#### Role of the Classification Token

The first-token state $F_{\mathrm{out},\mathrm{cls}}$ is not a summary of the patch sequence. It is initialized from a learnable token and a learnable positional embedding, is excluded from LGAM local aggregation and from RFCA serialization and recalibration, and during cross-stream interaction exchanges information only with the corresponding CLS state of the other stream. Because both state-space branches retain causal token dependencies and the CLS token is prepended, no patch token can influence it through those branches. For a trained model, this state therefore does not depend on the input point cloud and acts approximately as a learned reference or bias-like component when concatenated with the pooled patch descriptor. All input-dependent global information enters the classifier through $p_{\mathrm{global}}$, i.e., the sum of max and mean pooling over the fused patch tokens, and the CLS component is retained only for compatibility with the readout convention inherited from Mamba3D [11].

The classification head $H_{\mathrm{cls}}$ contains two 256-dimensional hidden layers, each followed by BatchNorm, ReLU, and dropout with rate 0.5, before the final classifier. Classification is optimized with the cross-entropy objective and the label-smoothing setting specified in the experimental configuration. For point-wise prediction tasks, the unified representation is passed to the ShapeNetPart decoder used in the corresponding experimental protocol.

### Appendix A.7. ShapeNetPart Configuration and Decoder

#### Appendix A.7.1. Encoder Configuration and Feature Extraction

The segmentation encoder contains four stages with feature dimension d=384. The input point cloud is grouped around 128 centers selected by farthest point sampling, with 32 nearest neighbors per patch. The decoder reads only the fourth and final stage, corresponding to the zero-based extraction index 3; features from earlier stages are not concatenated.

The segmentation RFCA branch operates on the original patch order without Morton serialization. Its patch-processing path uses a standard one-dimensional convolution with kernel size 7 and 384 input and output channels, followed by BatchNorm and SiLU. Channel attention uses global average pooling and a 384→96→384 mapping with ReLU and hard-sigmoid activations. Position attention uses channel-wise average and maximum responses, followed by a kernel-size-7 convolution and a sigmoid activation. The recalibrated patch features are subsequently processed by LayerNorm and SiLU.

After the final cross-stream interaction, the two states are fused using a token-wise gate. Let $X_{g},X_{s}\in R^{(G+1)\times d}$ denote these states, where G=128 and the first token is CLS. The fused patch representation is

(A42) $$\begin{array}{cc} a\; & =\sigma \;\left(\psi \;\left({\left[X_{g},X_{s}\right]}_{\mathrm{ch}}\right)\right),\\ F_{p}\; & =\mathrm{LN}\;\left({\left[a⊙X_{g}+\left(1-a\right)⊙X_{s}\right]}_{p}\right)\in R^{128\times 384}. \end{array}$$

Here, ψ is a 768→384→1 MLP with GELU between its linear layers, and ${\left[\;\cdot \;\right]}_{p}$ removes the CLS token. The scalar gate is broadcast over feature channels. The decoder therefore uses normalized fused patch features, without directly including the CLS state.

#### Appendix A.7.2. Feature Interpolation

Patch features are propagated directly from the 128 patch centers to the original input points using three-nearest-neighbor interpolation. For input point $p_{i}$, let $N_{3}\left(i\right)$ denote its three nearest patch centers. The interpolated feature is

(A43) $${\tilde{f}}_{i}=\sum_{j\in N_{3}\left(i\right)}w_{ij}F_{p,j},\;w_{ij}=\frac{(∥p_{i}-c_{j}{{∥}_{2}^{2}+\epsilon )}^{-1}}{\sum_{k\in N_{3}\left(i\right)}(∥p_{i}-c_{k}{{∥}_{2}^{2}+\epsilon )}^{-1}},\;\epsilon =10^{-8}.$$

The 384-dimensional interpolated feature is concatenated with the three input coordinates. A shared point-wise MLP with channel dimensions 387→1536→1024 then produces the propagated point feature $h_{i}$. Both layers use 1×1 convolutions, BatchNorm, and ReLU.

#### Appendix A.7.3. Global Context and Category Conditioning

Global maximum and average pooling are applied separately over the patch dimension of $F_{p}$, producing two 384-dimensional descriptors:

(A44) $$g_{\max}=\mathrm{MaxPool}\left(F_{p}\right),\;g_{\mathrm{avg}}=\mathrm{MeanPool}\left(F_{p}\right).$$

The ground-truth object category is supplied during both training and evaluation as a 16-dimensional one-hot vector. A bias-free 1×1 convolution maps this vector to a 64-dimensional category embedding $e_{c}$, followed by BatchNorm and LeakyReLU with negative slope 0.2. The two global descriptors and the category embedding are broadcast to all input points.

#### Appendix A.7.4. Point-Wise Prediction and Training Objective

The decoder concatenates the propagated point feature, both global descriptors, and the category embedding:

(A45) $$z_{i}=\left[h_{i};g_{\max};g_{\mathrm{avg}};e_{c}\right]\in R^{1856}.$$

The prediction head uses three 1×1 convolutions with 1856, 512, 256, and *Q* channels for the successive layers, where *Q* is the number of output part classes. The first two layers are followed by BatchNorm and ReLU, and dropout with probability 0.5 is applied after the first activation. Log-softmax is applied over the part classes, and training uses point-wise negative log-likelihood loss, equivalent to cross-entropy on the corresponding logits.

### Appendix A.8. Masked Reconstruction Pre-Training

#### Appendix A.8.1. Data and Masking

Pre-training uses the ShapeNet-55 training split, with the provided test split retained for held-out evaluation. No new dataset split is constructed, and category labels are not used in the reconstruction objective. Each input point cloud contains 1024 points and is partitioned into 64 patches, each containing 32 points. For each training sample, patches are randomly selected for masking with a masking ratio of 0.6. The encoder processes the visible patch tokens together with the learnable CLS token; masked patch tokens are excluded from the encoder input.

#### Appendix A.8.2. Reconstruction Objective

The complete dual-stream encoder is optimized jointly under the masked reconstruction objective. The encoded visible features and mask tokens are supplied to the reconstruction decoder to predict the points of the masked patches. Training minimizes the Chamfer-L2 distance between the predicted and ground-truth point sets of the masked patches.

#### Appendix A.8.3. Transfer to Downstream Tasks

After pre-training, the reconstruction decoder is discarded, and the pretrained encoder weights are transferred to the downstream ORE-Mamba model. LGAM and RFCA inherit the parameters of their corresponding pretrained modules, and the pretrained CLS token is retained. A task-specific prediction head is randomly initialized. The encoder and prediction head are then fine-tuned jointly in an end-to-end manner. The optimization settings are summarized in Table A2.

**Table A2:** Training configurations for ShapeNet-55 pre-training and downstream evaluation on ScanObjectNN, ModelNet40, and ShapeNetPart.

Setting	Pre-Training ShapeNet-55	ScanObjectNN	ModelNet40	ShapeNetPart
Input points	1024	2048	1024	2048
Number of patches *M*	64	128	64	128
Points per patch $K_{p}$	32	32	32	32
Optimizer	AdamW	AdamW	AdamW	AdamW
Initial learning rate	$1\times 10^{-3}$	$5\times 10^{-4}$	$3\times 10^{-4}$	$2\times 10^{-4}$
Weight decay	0.05	0.05	0.05	0.05
Label smoothing ϵ	N/A	0	0	0
Batch size	128	16	32	16
Training epochs	300	300	300	300
Learning-rate scheduler	Cosine	Cosine	Cosine	Cosine
Warm-up/initial stage	10 epochs	10 epochs	10 epochs	10 epochs
Random seed(s)	0	0, 1, 2	0, 1, 2	0, 1, 2

### Appendix A.9. Training Configuration and Model Selection

Table A2 summarizes the configurations for pre-training on ShapeNet-55, object classification on ScanObjectNN and ModelNet40, and part segmentation on ShapeNetPart. The input point count and number of patch tokens depend on the benchmark, while each patch contains 32 points.

For object classification on ScanObjectNN and ModelNet40, the cosine learning-rate scheduler is updated once per epoch with a configured scheduling period of 300 epochs. The minimum learning rate and the warm-up initial learning rate are both $1\times 10^{-6}$.

### Appendix A.10. Few-Shot Evaluation Protocol

#### Appendix A.10.1. Task Construction and Class Selection

We evaluate 5-way 10-shot, 5-way 20-shot, 10-way 10-shot, and 10-way 20-shot recognition on ModelNet40. Each configuration contains 10 predefined tasks, indexed from 0 to 9. These are separate task splits rather than 10-fold cross-validation partitions.

For each *N*-way *K*-shot task, *N* categories are randomly selected from the 40 ModelNet40 categories. For each selected category, *K* objects are sampled from the original training set to form the support set, and 20 objects are sampled from the original test set to form the query set. The support and query sets therefore contain NK and 20N objects, respectively. They share the same selected categories but contain different objects. Task labels are remapped to 0,…,N−1. The predefined splits are loaded once and kept fixed throughout adaptation. Predictions are obtained by taking the argmax over all 40 logits.

There is no additional fixed, class-disjoint base/novel partition. Categories may overlap across different tasks. Accordingly, this protocol evaluates adaptation from limited labeled examples within sampled ModelNet40 categories.

#### Appendix A.10.2. Adaptation Procedure

A separate model is constructed for each task. In the pretrained setting, each task starts from the same designated pretrained encoder checkpoint, with a newly initialized classification head. The encoder and classification head are jointly updated using supervised classification loss on the support set. The encoder is not frozen.

The implementation retains a 40-output classification head while using the remapped task labels. Adaptation uses AdamW with an initial learning rate of $5\times 10^{-4}$, weight decay of 0.05, batch size of 32, cosine learning-rate scheduling, and a 10-epoch warm-up. The configured maximum training duration is 150 epochs. Label smoothing is disabled.

#### Appendix A.10.3. Evaluation and Model Selection

The model is repeatedly evaluated on the fixed query set during training, without test-time voting. Gradient-based parameter updates use only the labeled support set. Query labels are used to compute evaluation accuracy and select the best-performing training epoch; they are not used for gradient-based parameter updates. The highest query-set accuracy observed during training is recorded for each split, and training terminates early if this accuracy reaches 100%. Thus, the query set participates in model selection and is not an independent final test set.

For each way–shot configuration, the reported mean and standard deviation are computed over the 10 task-level best query accuracies. These statistics summarize variation across the task runs, including their category selections, support/query samples, and model training. They are not statistics across three random seeds for a single fixed task.

Because the query set is also used for model selection, it does not constitute an independent final test set. Selecting the best query accuracy can produce optimistic performance estimates. We therefore interpret these results within this specific evaluation protocol rather than as an unbiased estimate of transfer to unseen categories.

### Appendix A.11. Detailed Complexity Derivation

This subsection provides the module-level derivations underlying the complexity analysis in Section 3.6. Let *M* denote the number of patch tokens, T=M+1 the total number of tokens, *d* the embedding dimension, and *L* the number of evolution stages. We further use $k_{g}$ for the LGAM neighborhood size, $d_{m}$ for the projected state-space mixing dimension, $k_{m}$ for the branch-wise convolution width, $n_{s}$ for the state dimension, κ for the RFCA receptive-field expansion factor, and $d_{r}$ for the RFCA bottleneck width.

We count leading-order arithmetic, comparison, and indexing costs. Bias addition, element-wise activation, normalization, residual addition, permutation indexing, and broadcasting are omitted when they are lower-order terms.

#### Appendix A.11.1. LGAM

##### Patch-Center Neighborhood Construction

The current implementation constructs the patch-center neighborhoods from the full pairwise Euclidean distance matrix. Its complexity is

(A46) $$C_{\mathrm{knn}}=O\left(M^{2}\right).$$

For a fixed neighborhood size $k_{g}$, extracting the nearest neighbors does not change the leading order of the pairwise-distance computation.

##### Local Geometry Aggregation

For the $Mk_{g}$ center–neighbor relations, the edge projection from 2d+3 input channels to 2d output channels requires

(A47) $$C_{\mathrm{edge}}=Mk_{g}\left(2d+3\right)\left(2d\right).$$

Neighbor-wise maximum aggregation contributes $O(Mk_{g}d)$, while projecting the pooled 2d-dimensional response back to *d* channels requires

(A48) $$C_{\mathrm{local},\mathrm{out}}=2Md^{2}.$$

Consequently, the learnable local geometry aggregation has complexity

(A49) $$\begin{array}{cc} C_{\mathrm{LGA}}\; & =O\left(Mk_{g}d^{2}+Mk_{g}d+Md^{2}\right)\\ \; & =O\left(Mk_{g}d^{2}\right), \end{array}$$

where the second equality follows from $k_{g}\geq 1$ and the dominance of the channel projections.

##### Projected-Channel State-Space Mixer

The shared input projection maps each *d*-dimensional token to a $2d_{m}$-dimensional joint content–gate representation and therefore costs $2Tdd_{m}$. After content–gate decomposition, the two branch-specific convolutions and selective state-space scans contribute

(A50) $$O\left(2Td_{m}\left(k_{m}+n_{s}\right)\right).$$

The shared output projection from $d_{m}$ to *d* channels requires $Td_{m}d$. After absorbing constant factors, the complete mixer complexity is

(A51) $$C_{\mathrm{mixer}}=O\left(Tdd_{m}+Td_{m}\left(k_{m}+n_{s}\right)\right).$$

Combining neighborhood construction, local geometry aggregation, and projected-channel state-space mixing gives

(A52) $$C_{\mathrm{LGAM}}=O\left(M^{2}+Mk_{g}d^{2}+Tdd_{m}+Td_{m}\left(k_{m}+n_{s}\right)\right).$$

#### Appendix A.11.2. RFCA

##### Morton Serialization

Morton-code construction is linear in the number of patch centers, whereas sorting the codes requires

(A53) $$C_{\mathrm{sort}}=O\left(M\log M\right).$$

The subsequent permutation and inverse-permutation indexing require O(Md) operations and are lower order than the major channel projections.

##### Receptive-Field Expansion

The depthwise one-dimensional convolution produces κd output channels, with each output channel applying a kernel of width κ over *M* serialized positions. Its complexity is

(A54) $$C_{\mathrm{dwconv}}=M\kappa^{2}d.$$

Constructing the serialized-position and expanded-channel descriptors, as well as applying the resulting broadcast recalibration, contributes O(Mκd).

##### Position–Channel Attention and Output Projection

With bottleneck width $d_{r}$, the shared context transformation and the two attention heads require

(A55) $$O\left(\left(M+\kappa d\right)d_{r}\right).$$

The output projection from the expanded dimension $d_{e}=\kappa d$ to the original embedding dimension requires

(A56) $$C_{\mathrm{rfca},\mathrm{out}}=M\kappa d^{2}.$$

The complete RFCA complexity is therefore

(A57) $$\begin{array}{cc} C_{\mathrm{RFCA}}=O(\; & M\log M+M\kappa^{2}d+M\kappa d\\ \; & +\left(M+\kappa d\right)d_{r}+M\kappa d^{2}). \end{array}$$

Since the output channel projection is the dominant learned transformation, this expression can be summarized as

(A58) $$C_{\mathrm{RFCA}}=O\left(M\log M+M\kappa^{2}d+M\kappa d^{2}\right).$$

#### Appendix A.11.3. Interaction and Fusion

Each cross-stream direction contains two token-wise linear transformations from *d* to *d* channels. The two direction-specific projections therefore require

(A59) $$\begin{array}{cc} C_{\mathrm{inter}}\; & =4Td^{2}+O\left(Td\right)\\ \; & =O(Td^{2}) \end{array}$$

at each evolution stage.

The final gate predictor follows a 2d→d→1 architecture. Its complexity is

(A60) $$\begin{array}{cc} C_{\mathrm{fusion}}\; & =2Td^{2}+O\left(Td\right)\\ \; & =O(Td^{2}). \end{array}$$

The element-wise convex combination of the two streams contributes only O(Td) and is therefore lower order.

#### Appendix A.11.4. Overall Encoder

The per-stage computation consists of LGAM, RFCA, and bidirectional cross-stream interaction:

(A61) $$C_{\mathrm{stage}}=C_{\mathrm{LGAM}}+C_{\mathrm{RFCA}}+C_{\mathrm{inter}}.$$

Combining the *L* evolution stages with the final adaptive fusion gives

(A62) $$C_{\mathrm{encoder}}=LC_{\mathrm{stage}}+C_{\mathrm{fusion}}.$$

Substituting the component terms yields

(A63) $$\begin{array}{cc} C_{\mathrm{encoder}}=O(\; & L[M^{2}+Mk_{g}d^{2}+Tdd_{m}\\ \; & \;+Td_{m}\left(k_{m}+n_{s}\right)+M\log M\\ \; & \;+M\kappa^{2}d+M\kappa d^{2}+Td^{2}]\\ \; & \;+Td^{2}). \end{array}$$

Since

(A64) $$T=M+1=\Theta \left(M\right),\;d_{m}=\Theta \left(d\right),$$

and $k_{g}$, $k_{m}$, $n_{s}$, and κ are fixed architectural constants, the current implementation can be summarized as

(A65) $$\begin{array}{cc} C_{\mathrm{encoder}}\; & =O\left(L\left[M^{2}+M\log M+Md^{2}\right]+Md^{2}\right)\\ \; & =O\left(L\left[M^{2}+M\log M+Md^{2}\right]\right). \end{array}$$

The final $Md^{2}$ term in the first line corresponds to adaptive fusion, which is executed only once after the final evolution stage and is absorbed by the stage-wise $LMd^{2}$ term in the second line.

##### Cached Geometric Indexing

In the current implementation, patch-center KNN and Morton sorting are recomputed at every evolution stage. However, both operations depend only on the patch-center coordinates, which remain fixed throughout the encoder. Their indices can therefore be computed once per forward pass and reused by all *L* stages. Under this cached implementation, the complexity becomes

(A66) $$\begin{array}{cc} C_{\mathrm{encoder}}^{\mathrm{cached}}\; & =O\left(M^{2}+M\log M+LMd^{2}+Md^{2}\right)\\ \; & =O\left(M^{2}+M\log M+LMd^{2}\right). \end{array}$$
